# New species and distributional records of Aleocharinae (Coleoptera, Staphylinidae) from Ontario, Canada, with a checklist of recorded species

**DOI:** 10.3897/zookeys.186.2947

**Published:** 2012-04-26

**Authors:** Adam J. Brunke, Jan Klimaszewski, Julie-Anne Dorval, Caroline Bourdon, Steven M. Paiero, Stephen A. Marshall

**Affiliations:** 1 Zoological Museum (Natural History Museum of Denmark), 15 Universitetsparken, University of Copenhagen, Copenhagen, DK 2100; 2 Natural Resources Canada, Canadian Forest Service, Laurentian Forestry Centre, 1055 du P.E.P.S., P.O. Box 10380, Stn. Sainte-Foy, Québec, Quebec, Canada G1V 4C7; 3 Cégep de Sainte-Foy, 2410, chemin Sainte-Foy, Québec, Quebec, Canada G1V 1T3; 4 University of Guelph Insect Collection and Insect Systematics Laboratory, 1216 Edmund C. Bovey Building, School of Environmental Sciences, University of Guelph, Guelph, Ontario, N1G 2W1, Canada

**Keywords:** Canada, Ontario, biodiversity, taxonomy, distributional records, Aleocharinae

## Abstract

The Aleocharinae (Coleoptera: Staphylinidae) of Ontario were reviewed in the context of recently studied material, primarily from insect surveys conducted by the University of Guelph Insect Collection (Ontario, Canada). *Aleochara daviesi* Klimaszewski & Brunke **sp. n.**, *Agaricomorpha websteri* Klimaszewski & Brunke **sp. n.**, *Atheta (Microdota) alesi* Klimaszewski & Brunke **sp. n.**, *Dinaraea backusensis* Klimaszewski & Brunke **sp. n.**, and *Strigota obscurata* Klimaszewski & Brunke **sp. n.** are described as new to science. We also report 47 new Ontario records and 24 new Canadian records. *Callicerus rigidicornis* (Erichson) and *Alevonota gracilenta* (Erichson) are newly reported from North America as adventive species. A checklist, with Canadian distributions by province, of the 224 species of Aleocharinae known from Ontario is given. The following species are placed in subjective synonymy with *Dexiogyia angustiventris* (Casey): (*Dexiogyia asperata* (Casey) **syn. n.**, *Dexiogyia abscissa* (Casey) **syn. n.**, *Dexiogyia tenuicauda* (Casey) **syn. n.**, *Dexiogyia intenta* (Casey) **syn. n.**, *Dexiogyia alticola* (Casey) **syn. n.**). The following species are placed in subjective synonymy with *Acrotona subpygmaea* (Bernhauer): (*Acrotona avia* (Casey) **syn. n.**, *Acrotona puritana* (Casey) **syn. n.**). Lectotypes are designated for *Thiasophila angustiventris* Casey, *Thiasophila asperata* Casey, *Ischnoglossa intenta* Casey, *Oxypoda rubescans* Casey, *Chilopora americana* Casey, *Chilopora fuliginosa* Casey, *Coprothassa smithi* Casey, *Atheta subpygmaea* Bernhauer, *Colpodota puritana* Casey, *Strigota seducens* Casey, *Trichiusa compacta* Casey, *Trichiusa hirsuta* Casey and *Trichiusa robustula* Casey.

## Introduction

Over the past ten years, the Aleocharinae (Coleoptera: Staphylinidae) have been one of the most active areas of beetle systematics research in Canada (see references in Gouix and Klimaszewski (2007)), yet knowledge of their true diversity is still rather fragmentary. Focused studies on the aleocharine fauna of the Maritime Provinces of Canada (e.g. Majka and Klimaszewski 2008a; Webster et al. 2009; Majka and Klimaszewski 2010), Newfoundland (Klimaszewski et al. 2011) and Yukon Territory (Klimaszewski et al. 2008a; Klimaszewski et al. 2012) have resulted in the discovery of many new species and have approximately doubled (Maritimes, Yukon) or more than quadrupled (Newfoundland) the known diversity in these areas since the publication of the most recent catalog of Canadian Aleocharinae (Gouix and Klimaszewski 2007).

The aleocharine fauna of Ontario was summarized by Campbell and Davies (1991) (60 species) and updated by Gouix and Klimaszewski (2007) (108 species). Since then, revisions and faunistic studies of Canadian Aleocharinae have raised that total to its current state at 146 species. Recently we have had the opportunity to study new material from Ontario made available through biodiversity surveys by the University of Guelph Insect Collection, and select material deposited in other collections. We herein report the discovery of several new species, the taxonomic or diagnostic clarification of others and new records for Ontario, Canada and North America. Photographs of the habitus, genitalia and other relevant characters are provided to aid in their identification.

## Materials and methods

Specimens were examined using Wild Heerbrugg M5A and Nikon SMZ 1000 stereomicroscopes, and nearly all were dissected to examine features of the genitalia. In many cases, tergite and sternite 8 were also examined. These structures were dehydrated in absolute alcohol and mounted in Canada balsam on celluloid microslides and pinned with the specimens from which they originated. Photographs were taken using an image processing system (Nikon SMZ 1500 stereoscopic microscope, Nikon Digital Camera DXM 1200F, and Adobe Photoshop software). Habitus photographs of all included species are provided, while genitalia are illustrated only for those species whose genitalia have not been shown in recent publications. Maps of each species’ distribution in Ontario, Canada were prepared using ARC MAP and Adobe Photoshop. In the species accounts, distributions are given by province or state (Canada, U.S.A.) or by country (elsewhere). These territories are abbreviated using Canada Post and United States Postal Service standards.

Morphological terminology mainly follows that used by Seevers (1978) and Ashe in Newton et al. (2000). The ventral (=parameral) part of the median lobe of the aedeagus is considered to be the part of the bulbus containing the foramen mediale, the entrance of the ductus ejaculatorius, and the adjacent venter of the tubus; the opposite side is referred to as the dorsal (=abparameral) part.

Material was examined from the following collections:

**CNC **Canadian National Collection of Insects, Ottawa, Ontario, Canada

**DEBU **University of Guelph Insect Collection, Guelph, Ontario, Canada

**FMNH **Field Museum of Natural History, Chicago, Illinois, USA

**LFC **Laurentian Forestry Centre, Quebec, Quebec, Canada

**MZLU **Museum of Lund University, Lund, Sweden

**NMNH **National Museum of Natural History, Smithsonian Institution, Washington D.C., USA

**ZMB **Museum für Naturkunde, Invalidenstrasse 43, 10115, Berlin, Germany

**ZMUC **Zoological Museum, University of Copenhagen, Copenhagen, Denmark

**RWC **Reginald Webster Collection, Charters Settlement, New Brunswick, Canada

Additionally, distributions of species included in this account were kindly checked by A. Davies (CNC) against his database of Canadian Staphylinidae to be published in the upcoming second edition of the ‘Checklist of Beetles of Canada and Alaska’ (Davies in Bousquet et al. in prep.). Distributions marked herein with an asterisk (*) represent records based entirely on these data. In the species accounts, the number of specimens for each collection event is given directly preceding the collection abbreviation in brackets. Where appropriate, short discussions pertaining to individual species are given in the species accounts under ‘comments’. We follow the higher taxonomic organization of Ashe in Newton et al. (2000) with changes reflected in Gouix and Klimaszewski (2007) and Paśnik (2010).

## Results

As a result of the present study we recognize 224 species of Aleocharinae in Ontario. A checklist by tribe of all known Ontario Aleocharinae species and their distributions in Canada is given in Table 1. *Aleochara daviesi* Klimaszewski & Brunke sp. n., *Agaricomorpha websteri* Klimaszewski & Brunke sp. n., *Atheta (Microdota) alesi* Klimaszewski & Brunke sp. n., *Dinaraea backusensis* Klimaszewski & Brunke sp.n., and *Strigota obscurata* Klimaszewski & Brunke sp. n. are described as new to science. Twenty-four species are newly recorded from Canada, 47 species are newly recorded from Ontario, and the Palaearctic species *Alevonota gracilenta* (Erichson, 1839) and *Callicerus rigidicornis* (Erichson, 1839) are newly reported as introduced to North America. The genera *Agaricomorpha* Ashe, 1984, *Alevonota* Thomson, 1858, *Callicerus* Gravenhorst, 1802, *Dexiogyia* Thomson, 1858, *Phanerota* Casey 1906, *Stethusa* Casey, 1910 and *Thecturota* Casey, 1894 are new for the Canadian fauna.

**Table 1.** Species of Aleocharinae recorded from Ontario and their provincial and territorial distribution within Canada. Provinces in bold denote new records given in the present publication. Additional records provided by A. Davies (see Methods) are marked by *.

Tribe Gymnusini	
*Gymnusa atra* Casey	YT, NT, NU, BC, AB, MB, ON, QC, NB, NS, NL
*Gymnusa campbelli* Klimaszewski	YT, NT, MB*, ON, QC, NB, NL
*Gymnusa grandiceps* Casey	MB, ON, QC, NB, NS, NL
*Gymnusa pseudovariegata* Klimaszewski	YT, NT, BC, AB, MB, ON, QC, NB, NS, NL
*Gymnusa smetanai* Klimaszewski	YT, NT, MB, ON, NL
Tribe Deinopsini	
*Deinopsis canadensis* Klimaszewski	ON, NB, NL
*Deinopsis harringtoni* Casey	MB*, ON, QC, NB, NS, NL
*Deinopsis illinoisensis* Klimaszewski	**ON**
*Deinopsis rhadina* Klimaszewski	ON, QC*, NB
Tribe Aleocharini	
*Aleochara assiniboin* Klimaszewski	YT, BC, SK, MB, ON
*Aleochara bilineata* Gyllenhal †	BC, AB, SK*, MB, ON, QC, NB, NS, PE, NL
*Aleochara bimaculata* Gravenhorst	NT, BC, AB, SK, MB, ON, QC, NB, NS, NL
*Aleochara castaneipennis* Mannerheim	YT, NT*, BC, AB, ON, QC, NB, NS, NL
*Aleochara curtula* (Goeze) †	BC, ON, QC, NB, NS, PE, NL
*Aleochara daviesi* Klimaszewski & Brunke, sp.n.	**ON**
*Aleochara fumata* Gravenhorst †	YT, BC, AB, MB, ON, QC, NB, NS, PE, NL
*Aleochara gracilicornis* Bernhauer	NT, BC, SK, MB, ON, QC, NB, NS
*Aleochara inexpectata* Klimaszewski	ON, QC, NB, NS
*Aleochara lacertina* Sharp	BC, AB, SK, MB, ON, QC, NB, NS, NL
*Aleochara lanuginosa* Gravenhorst †	BC, AB, ON, QC, NB, NS, NL
*Aleochara lata* Gravenhorst†	YT, BC, SK, MB, ON, QC
*Aleochara lustrica* Say	**ON**
*Aleochara ocularis* Klimaszewski	MB, ON, QC
*Aleochara rubricalis* (Casey)	BC, ON (doubtful record)
*Aleochara rubripennis* (Casey)	MB, ON, QC, NB
*Aleochara sculptiventris* (Casey)	ON, QC, NB, NL
*Aleochara sekanai* Klimaszewski	YT, NT*, AB, MB, ON, NL
*Aleochara speculicollis* Bernhauer	AB, ON, QC
*Aleochara tahoensis* Casey	YT, NT, BC, AB, SK, MB, ON, NB, NS
*Aleochara thoracica* Casey	ON, QC, NB, NL
*Aleochara tristis* Gravenhorst†	**ON,** QC, NB, NL
*Aleochara verna* Say	YT, BC, AB, SK, MB, ON, QC, NB, NS, PE, NL
*Tinotus caviceps* Casey	ON, QC
*Tinotus morion* (Gravenhorst) †	BC, AB, ON, QC, NB, NS, NL
*Tinotus trisectus* Casey	**ON**
Tribe Hoplandriini	
*Hoplandria klimaszewskii* Génier	**ON,** QC
*Hoplandria laevicollis* (Notman)	**ON,** QC
*Hoplandria laeviventris* Casey	**ON**
*Hoplandria lateralis* (Melsheimer)	ON, QC, NB
*Platandria carolinae* Casey	**ON**
Tribe Oxypodini	
*Amarochara brevios* Assing	**ON**
*Amarochara fenyesi* Blatchley	**ON**
*Calodera parviceps* (Casey)	YT, ON, NB, NS
*Crataraea suturalis* (Mannerheim) †	BC, SK, **ON,** NB, NS, NL
*Devia prospera* (Erichson)	YT, NT, BC, AB, MB, ON, NB, NL
*Dexiogyia angustiventris* (Casey)	**ON**
*Gennadota canadensis* Casey	ON, QC, NB, NS
*Hylota ochracea* Casey	NT, ON, QC, NB, NS
*Ilyobates bennetti* Donisthorpe †	**ON,** QC, NB, NS
*Meotica ‘pallens’* Redtenbacher †	BC, ON, NS
*Ocyusa canadensis* Lohse	YT, **ON**
*Oxypoda amica* Casey	YT, MB, ON, QC, NB, NS
*Oxypoda brachyptera* (Stephens) †	ON, QC, NB, NS, NL
*Oxypoda canadensis* Klimaszewski	YT, NT, AB, MB, ON, QC, NL
*Oxypoda chantali* Klimaszewski	ON, QC, NS
*Oxypoda convergens* Casey	AB, ON, QC, NB, NS, NL
*Oxypoda demissa* Casey	YT, ON, QC, NB, NS, NL
*Oxypoda frigida* Bernhauer	YT, NT, BC, AB, ON, QC, NB, NS, NL
*Oxypoda gnara* Casey	ON, QC, NB
*Oxypoda grandipennis* (Casey)	YT, BC, AB, ON, QC, NB, NS, NL
*Oxypoda hiemalis* Casey	YT, NT, ON, QC, NB, NS, NL
*Oxypoda lacustris* Casey	YT, NT, BC, AB, ON, QC, NB, NL
*Oxypoda lucidula* Casey	YT, NT, AB, MB, ON, QC, NB, NL
*Oxypoda opaca* (Gravenhorst) †	BC, ON, NB, NS, NL
*Oxypoda operta* Sjöberg	YT, AB, ON, QC, NS, NL
*Oxypoda orbicollis* Casey	YT, AB, ON, QC, NB, NS, NL
*Oxypoda perexilis* Casey	ON, QC, NS
*Oxypoda pseudolacustris* Klimaszewski	AB, ON, QC, NB, NS, NL
*Oxypoda rubescans* Casey	**ON**
*Oxypoda vockerothi* Klimaszewski	ON, NB
*Parocyusa americana* (Casey)	**ON**
*Parocyusa fuliginosa* (Casey)	**ON,** NL
*Phloeopora arctica* Lohse	YT, NT, ON
Tribe Tachyusini	
*Brachyusa helenae* (Casey)	YT, NT, **ON,** NB,NL
*Gnypeta caerulea* (Sahlberg)	YT, NT, BC*, AB, SK, MB, ON, QC, NB, NS, PE, NL
*Gnypeta canadensis* Klimaszewski	AB, ON
*Gnypeta carbonaria* (Mannerheim)	NT, AB, SK, MB, ON, QC, NB, NL
*Gnypeta helenae* Casey	BC, AB, **ON**
*Gnypeta nigrella* (LeConte)	**ON,** NB, NL
*Meronera venustula* (Erichson)	ON, QC, NB
*Tachyusa americana* Casey	ON, QC, NB
*Tachyusa americanoides* Paśnik	NT, BC, AB, MB, ON, QC, NB, NS, NL
Tribe Hypocyphtini	
*Cypha inexpectata* Klimaszewski & Godin	YT, ON
Tribe Myllaenini	
*Myllaena arcana* Casey	AB, ON, QC, NB, NS, NL
*Myllaena audax* Casey	NT, BC, ON, QC, NB, NL
*Myllaena cuneata* Notman	**ON,** NS
*Myllaena insomnis* Casey	YT, NT, BC, AB, SK, MB, ON, QC, NB, NS, NL
*Myllaena lucidificans* Casey	ON, QC, NB
*Myllaena potawatomi* Klimaszewski	**ON**
*Myllaena vulpina* Bernhauer	ON, NB, NS
Tribe Autaliini	
*Autalia rivularis* (Gravenhorst)	BC, AB, ON, QC, NB, NS, NL
Tribe Homalotini	
*Agaricomorpha websteri* Klimaszewski & Brunke, sp. n.	**ON, QC, NB, NS**
*Eumicrota corruscula* (Erichson)	**ON,** QC, NB
*Eumicrota socia* (Erichson)	**ON,** QC, NB, NS, PE
*Euvira micmac* Klimaszewski & Majka	**ON,** NB, NS
*Gyrophaena affinis* Mannerheim †	BC, MB, **ON,** QC, NB, NS, NL
*Gyrophaena antennalis* Casey	**ON,** NB, NS, NL
*Gyrophaena brevicollis* Seevers	**ON**
*Gyrophaena caseyi* Seevers	**ON,** QC
*Gyrophaena criddlei* Casey	YT? MB, **ON,** NB, NL
*Gyrophaena dybasi* Seevers	**ON,** NB
*Gyrophaena egena* Casey	ON, QC
*Gyrophaena flavicornis* Melsheimer	ON, QC, NB, NS
*Gyrophaena fuscicollis* Casey	**ON,** NB
*Gyrophaena gaudens* Casey	ON, QC, NB, PE
*Gyrophaena gilvicollis* Casey	**ON,** NB
*Gyrophaena insolens* Casey	MB*,ON, NB, NL
*Gyrophaena keeni* Casey	YT, BC, AB, ON, QC, NB, NL
*Gyrophaena meduxnekeagensis* Klimaszewski & Webster	**ON,** QC, NB
*Gyrophaena modesta* Casey	AB*, **ON,** NB, NS, NL
*Gyrophaena nana* (Paykull)	YT, BC, AB, MB, ON, NB, NL
*Gyrophaena nanoides* Seevers	ON, QC, NB, NL
*Gyrophaena neonana* Seevers	YT, **ON,** NB,NL
*Gyrophaena stroheckeri* Seevers	**ON**
*Gyrophaena subnitens* Casey	MB, ON, NB, NS
*Gyrophaena uteana* Casey	BC, AB*, **ON,** QC, NB
*Gyrophaena vitrina* Casey	ON, QC, NB, PE
*Homalota plana* (Gyllenhal) †	ON, NB, NS, NL
*Leptusa brevicollis* Casey	ON, QC, NB, NS, PE, NL
*Leptusa canonica* Casey	ON, QC, NB, NS, NL
*Leptusa carolinensis* Pace	**ON,** QC, NB, NS
*Leptusa cribulata* (Casey)	ON, QC
*Leptusa elegans* Blatchley	ON, QC
*Leptusa gatineauensis* Klimaszewski & Pelletier	BC, AB, ON, QC, NB, NS, NL
*Leptusa jucunda* Klimaszewski & Majka	ON, QC, NB, NS
*Leptusa opaca* Casey	ON, QC, NB, NS, PE, NL
*Neotobia alberta* Ashe	AB, MB, ON, QC, NB
*Phanerota fasciata* (Say)	**ON**
*Phymatura blanchardi* (Casey)	AB, **ON,** NB
*Silusa alternans* Sachse	ON, QC, NB, NS, PE
*Silusa californica* (Bernhauer)	YT, BC, AB, ON, QC, NB, NS, PE, NL
*Silusida marginella* (Casey)	ON, QC, NB, NS, PE, NL
*Thecturota pusio* (Casey)	**ON**
Tribe Placusini	
*Placusa canadensis* Klimaszewski	ON, QC, NS
*Placusa despecta* Erichson	ON, QC
*Placusa incompleta* Sjöberg	BC, **ON,** QC, NB, NS, NL
*Placusa pseudosuecica* Klimaszewski	BC, ON, QC
*Placusa tachyporoides* (Walt)	BC, ON, QC, NB, NS
*Placusa tacomae* Casey	YT, NT, BC, AB, ON, QC, NB, NS, NL
*Placusa vaga* Casey	YT, NT. BC, **ON,** QC, NB, NS
Tribe Athetini	
*Acrotona smithi* (Casey)	**ON,** NB
*Acrotona subpygmaea* (Bernhauer)	**ON,** NS
*Alevonota gracilenta* (Erichson) †	**ON**
*Aloconota sulcifrons* (Stephens) †	**ON,** QC, NB, NL
*Atheta aemula* (Erichson)	**ON,** QC, NB
*Atheta alesi* Klimaszewski and Brunke, sp. n.	**ON**
*Atheta annexa* Casey	ON, QC, NB, NS, NL
*Atheta borealis* Klimaszewski & Langor	**ON,** NL
*Atheta brunswickensis* Klimaszewski	YT, ON, NS
*Atheta burwelli* (Lohse)	YT, **ON,** QC, NB, NL
*Atheta campbelli* (Lohse)	YT, **ON,** NL
*Atheta capsularis* Klimaszewski	YT, **ON,** QC, NB, NL
*Atheta circulicollis* Lohse	**ON,** QC, NB, NL
*Atheta crenuliventris* Bernhauer	ON, QC, NB, NL
*Atheta dadopora* Thomson	YT, AB, ON, NB, NS, PE, NL
*Atheta districta* Casey	BC, AB, ON, NB, NS, NL
*Atheta festinans* (Erichson)	ON
*Atheta frosti* Bernhauer	BC, ON, QC, NB, NS, NL
*Atheta graminicola* (Gravenhorst)	YT, NT, BC, AB, MB, ON, QC, NB, NL
*Atheta hampshirensis* Bernhauer	BC, ON, QC, NB, NS, NL
*Atheta klagesi* Bernhauer	YT, BC, AB, ON, QC, NB, NS, PE, NL
*Atheta modesta* (Melsheimer)	AB, ON, QC, NB, NS
*Atheta nescia* (Casey)	BC, **ON**
*Atheta particula* (Casey)	**ON,** QC, NB, NS
*Atheta pennsylvanica* Bernhauer	ON, QC, NB, NS, NL
*Atheta platonoffi* Brundin	YT, BC, AB, ON, NB, NS, NL
*Atheta prudhoensis* (Lohse)	YT, ON, NB, NS, NL
*Atheta pseudocrenuliventris* Klimaszewski	YT, **ON,** NB, NS, NL
*Atheta pseudomodesta* Klimaszewski	ON, QC, NB, NS, NL
*Atheta remulsa* Casey	YT, BC, AB, ON, QC, NB, NS, NL
*Atheta savardae* Klimaszewski & Majka	**ON,** QC, NB, NS, NL
*Atheta strigosula* Casey	YT, BC, ON, QC, NB, NL
*Atheta terranovae* Klimaszewski & Langor	YT, **ON,** NB, NL
*Atheta ventricosa* Bernhauer	YT, BC, AB, ON, QC, NB, NS, NL
*Callicerus obscurus* Gravenhorst †	**ON**
*Callicerus rigidicornis* (Erichson) †	**ON**
*Clusiota impressicollis* (Bernhauer)	BC, ON, NB, NL
*Dalotia coriaria* (Kraatz)†	BC, AB, ON, NB, NS
*Dinaraea angustula* (Gyllenhal) †	YT, AB, ON, QC, NB, NS, PE, NL
*Dinaraea backusensis* Klimaszewki & Brunke, sp.n.	**ON**
*Earota dentata* (Bernhauer)	YT, BC, AB, MB, ON, QC, NB, NS, NL
*Hydrosmecta pseudodiosica* Lohse	YT, ON, NB
*Liogluta aloconotoides* Lohse	YT, ON, QC, NB, NS, NL
*Lypoglossa franclemonti* Hoebeke	YT, NT, AB, MB, ON, QC, NB, NS, NL
*Mocyta breviuscula* (Mäklin)	YT, BC, AB, **ON,** QC, NB, NS, NL
*Mocyta fungi* (Gravenhorst) †	YT, BC, AB, ON, QC, NB, NS, PE, NL
*Philhygra botanicarum* (Muona)	YT, BC, ON, NB, NS, NL
*Philhygra clemens* (Casey)	YT, BC, ON, QC, NB, NS
*Philhygra jarmilae* Klimaszewski & Langor	YT, **ON,** NB, NL
*Philhygra laevicollis* (Mäklin)	BC, **ON,** NB, NS
*Philhygra luridipennis* (Mannerheim)	**ON,** NB, NL
*Philhygra proterminalis* (Bernhauer)	**ON**
*Nehemitropia lividipennis* (Mannerheim) †	ON, QC, NB, NS, PE, NL
*Schistoglossa blatchleyi* (Bernhauer & Scheerpeltz)	YT, NT, MB, ON, QC, NB
*Schistoglossa brunswickensis* Klimaszewski & Webster	ON, QC, NB
*Seeversiella globicollis* (Bernhauer)	BC, AB, ON, QC, NS, NL
*Stethusa klimschi* (Bernhauer)	**ON**
*Stethusa spuriella* (Casey)	**ON**
*Strigota ambigua* (Erichson)	YT, **ON,** NS, PE, NL
*Strigota obscurata* Klimaszewski & Brunke, sp. n.	**ON**
*Strophogastra penicillata* Fenyes	AB, MB, ON, QC, NB, NS
*Thamiaraea brittoni* (Casey)	ON, QC, NB
*Trichiusa compacta* Casey	**ON**
*Trichiusa hirsuta* Casey	**ON**
*Trichiusa postica* Casey	ON, NS
*Trichiusa robustula* Casey	**ON**
Tribe Falagriini	
*Aleodorus bilobatus* (Say)	ON
*Aleodorus scutellaris* (LeConte)	ON
*Cordalia obscura* (Gravenhorst) †	ON, QC, NB, NS
*Falagria dissecta* Erichson	BC, AB, SK, MB, ON, QC, NB, NS
*Falagria sulcata* Paykull†	AB, ON, QC, NB
*Myrmecocephalus cingulatus* (LeConte)	ON, NS
Tribe Lomechusini	
*Drusilla canaliculata* (Fabricius) †	ON, QC, NB, NS, PE, NL
*Myrmecoecia lauta* (Casey)	ON
*Myrmedonota aidani* Maruyama & Klimaszewski	ON
*Pella carolinae* (Casey)	ON
*Pella gesneri* Klimaszewski	AB, MB, ON, NB
*Pella loricata* (Casey)	ON, NS
*Pella schmitti* (Hamilton)	ON, QC
*Platyusa sonomae* Casey	ON
*Xenodusa cava* (LeConte)	ON
*Xenodusa reflexa* (Walker)	BC, AB, SK, MB, ON, QC, NB, NS
*Zyras obliquus* (Casey)	BC, AB, MB, ON, QC, NB, NS, NL
*Zyras planifer* (Casey)	**ON**

†Considered adventive in North America.

## Systematic account of new species and distributional records

### Tribe Deinopsini Sharp, 1883

#### Deinopsis illinoisensis

Klimaszewski, 1979 New Canadian Record

http://species-id.net/wiki/Deinopsis_illinoisensis

**Figures 1–6.**
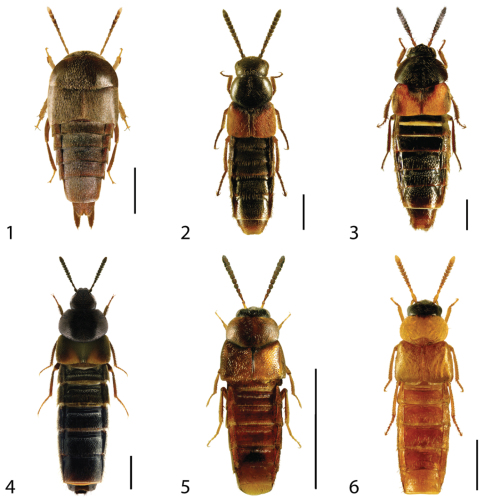
Dorsal habitus of: **1** *Deinopsis illinoisensis* Klimaszewski **2** *Aleochara daviesi* Klimaszewski & Brunke sp. n. **3** *Aleochara lustrica* Say **4** *Aleochara tristis* Gravenhorst **5** *Tinotus trisectus* Casey **6** *Hoplandria klimaszewskii* Génier. Scale 1mm.

**Maps 1–4.**
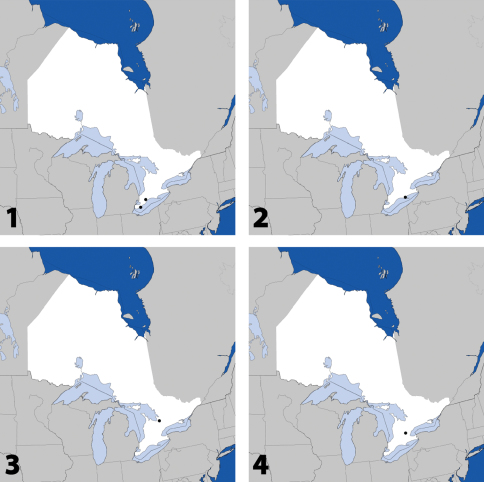
Distribution in Ontario of: **1** *Deinopsis illinoisensis* Klimaszewski **2** *Aleochara daviesi* Klimaszewski & Brunke sp. n. **3** *Aleochara lustrica* Say **4** *Aleochara tristis* Gravenhorst.

Fig. 1 Map 1 genitalia in Klimaszewski (1979)

##### Material examined.

CANADA: ON: *Chatham-Kent Co*.,Wheatley Provincial Park, treading at waters edge, 23.vii.2011, S.M. Paiero, 4 (DEBU); *Elgin Co*., Newport Forest, ~3km SW of Wardsville, 42°37'52"N, 81°46'43"W, 30.vii.2009, A. Brunke, 1 (DEBU).

##### Distribution.

Canada: ON; USA: CT, FL, IL, KY, LA, MA, MI, MS, OK, PA, TX (Klimaszewski 1979, Klimaszewski 1982a, Klimaszewski and Génier 1985, Klimaszewski and Frank 1992a). Native.

### Tribe Aleocharini Fleming, 1821

#### Aleochara (Echochara) daviesi

Klimaszewski & Brunke sp. n.

urn:lsid:zoobank.org:act:F542DCE5-7DB7-4F47-B16A-65C6189899D4

http://species-id.net/wiki/Aleochara_daviesi

Figs 2 80–82 Map 2

##### Type locality.

Canada, Ontario, Haldimand-Norfolk Reg., 6 km W of Saint Williams, Backus Woods, slough forest, 42°40'7"N, 80°29'34"W.

##### Type material.

Holotype (male): CANADA, ON:*Hald.-Norfolk Reg*., Backus Woods, North Block, 42°40'7"N, 80°29'34"W, 23.iv.2011, Brunke & Marshall, debu00340040 (DEBU).

##### Diagnosis.

Distinguished from other *Aleochara* by the following combination of characters: antennomere 4 subquadrate and 5–10 slightly transverse (Fig. 2); eyes extremely large, protruding laterally and close to frontal margin, postocular area of head about as large as eye in lateral view, postocular carina strong and complete; pronotum slightly transverse, with basal margin arcuate; elytra slightly longer than pronotum; abdomen subparallel for most of its length; basal metatarsomere slightly longer than the following tarsomere, tarsal claws exceptionally large, narrowly elongate; median lobe of aedeagus with large and narrowly elongate crista apicalis, tubus in lateral view swollen ventrally and sharply produced apically (Fig. 80). *Aleochara daviesi* is very similar externally to the western North American *Aleochara lobata* Klimaszewski from which it may be readily distinguished by the shape of the median lobe.

##### Description

**.** Body length 4.9 mm; black with legs, elytra (except narrowly at base) and abdominal tergites VII and VIII, rust brown; punctation of forebody coarse, dense and flattened, interspaces between punctures with fine meshed microsculpture (Fig. 2); head broadest apically with very short frons and with strong and complete postocular carina, pubescence of dorsal surface directed toward midline of disc, eyes extremely large, protruding laterally, and close to frontal margin of head, postocular area about as long as eye; antennae with antennomeres 1–3 elongate, antennomere 4 subquadrate and 5–10 slightly transverse; pronotum slightly transverse, shorter than elytra, pubescence directed obliquely posteriad from midline of disc, punctation flattened and forming transversely impressed line at base of disc; elytra with posterior margin nearly straight with slight lateral emargination, pubescence directed lateroposteriad from suture; abdomen subparallel for most of its length, tergites II-IV with deep and V with shallow impression, impressions with dense punctures separated from each other by a distance equal to or less than diameter of a puncture, punctures often touching; basal metatarsomere slightly longer than the following segment; tarsal claws exceptionally large, elongate and with surface smooth.

Male. Tergite 8 bicolored, dark brown/black basally and yellowish apically, truncate apically and with margin slightly crenulate (Fig. 81); sternite eight produced apically (Fig. 82); median lobe of aedeagus in lateral view with large and elongate bulbus produced ventrally at base, crista apicalis narrowly elongate and large, tubus swollen ventrally and sharply produced apically (Fig. 80).

Female: Unknown.

##### Distribution.

Presently known only from Backus Woods, an old growth deciduous forest in southern Ontario. *Aleochara daviesi* almost certainly occurs in the eastern United States and elsewhere in southern Canada.

##### Bionomics.

The holotype was collected by submerging forest litter near the margins of forest pools (some permanent). Other members of the subgenus *Echochara* are inhabitants of mammal burrows or caves (Klimaszewski 1984). As there are no cave systems at the type locality, we suspect that *Aleochara daviesi* occurs in the former situation. Although the staphylinids occurring in groundhog (*Marmota monax* (L.)) burrows have been sampled (Klimaszewski 1984, Smetana 1971, Smetana 1995, this paper) the fauna in burrows/nests of other mammals in eastern North America is essentially unknown. Future survey work in the nests of Nearctic moles, shrews and rodents is warranted.

##### Etymology.

This species is dedicated to our colleague Anthony Davies (CNC, Ottawa, Ontario, Canada) in recognition of his contribution to the knowledge of Canadian Staphylinidae and in appreciation of his assistance over the years in specimen loans, distributional records and curatorial matters, especially those relevant for this project.

#### Aleochara (Aleochara) lustrica

Say, 1832 New Canadian Record

http://species-id.net/wiki/Aleochara_lustrica

Fig. 3 Map 3 genitalia in Klimaszewski (1984)

##### Material examined.

CANADA: ON:*Simcoe Co*., Midhurst, 28.ix.2008, carrion, forest nr. Neretva St., A. Brunke, 1 (DEBU).

##### Distribution.

Canada: ON; USA: AL, AZ, AR, DC, FL, GA, IL, IN, KS, KY, LA, MD, MA, MI, MO, MS, NC, NH, NJ, NY, OH, OK, PA, SC, TN, TX, VA, WV, WI.Also known from Mexico and South America (Trinidad and Tobago) (Klimaszewski 1984, Klimaszewski and Génier 1987, Klimaszewski and Frank 1992c). Native.

#### Aleochara (Xenochara) tristis

Gravenhorst, 1806 New Ontario Record

http://species-id.net/wiki/Aleochara_tristis

Fig. 4 Map 4 genitalia in Klimaszewski (1984)

##### Material examined.

CANADA: ON:*Wellington Co*., Guelph, field, 20.ix.1984, Brian Wisenden, 1 (DEBU).

##### Distribution.

Canada: ON, QC, NB, NL; USA: CA, MN, NE, PA, VT. Widespread in Palaearctic, Oriental and Afrotropical Regions (Horion 1967; Moore and Legner 1975; Klimaszewski 1984; Klimaszewski and Cervenka 1986; Byers et al. 2000; Smetana 2004; Klimaszewski et al. 2005b; Klimaszewski et al. 2011). Adventive in Canada.

##### Comments.

*Aleochara tristis* was intentionally released in the United States in 1965 to control populations of Face Fly (*Musca autumnalis* DeGeer), a nuisance pest of and disease vector for livestock, which breeds in their dung (Jones 1967). In terms of biological control, the introduction appears to be a failure as this species is rarely collected and only as singletons. However, it is most certainly established in northeastern North America at low densities (Klimaszewski et al. 2005b, this study).

#### Tinotus trisectus

Casey, 1906 New Canadian Record

http://species-id.net/wiki/Tinotus_trisectus

**Maps 5–8.**
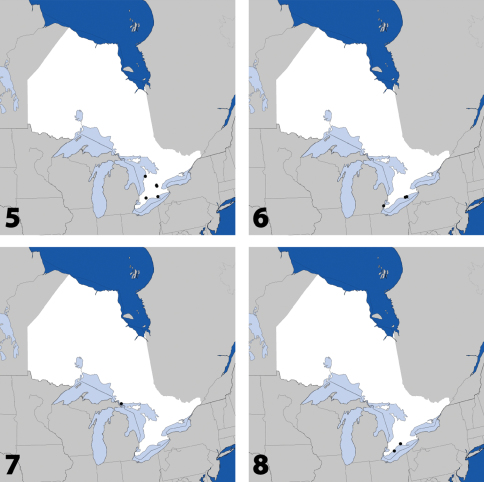
Distribution in Ontario of: **5** *Tinotus trisectus* Casey **6** *Hoplandria klimaszewskii* Génier **7** *Hoplandria laevicollis* (Notman) **8.** *Hoplandria laeviventris* Casey.

Fig. 5 Map 5 genitalia in Klimaszewski et al. (2002)

##### Material examined.

CANADA: ON:*Bruce Co*., Port Elgin, 15.vii.1980, P.F. Karrow, 1 (DEBU); *Chatham-Kent Co*., Glencoe, carrot field, pitfall, 17.v.2007, A. Brunke, 1 (DEBU); *Hald.-Norfolk Reg*., Turkey Point Prov. Park, site 2, 42°42'28"N, 80°20'29"W, savannah, at lights, 5.vii.2011, Brunke & Paiero, 1 (DEBU); *Wellington Co*., Guelph, Victoria Rd. & Conservation Line, soybean field, pitfall, 4.viii.2009, A. Brunke, 2 (DEBU), Guelph, woodland edge, 9.x.1991, C.S. Blanev, 1 (DEBU).

##### Distribution.

Canada: ON; USA: AZ, CA, ID, NY, OR, PA, TN (Klimaszewski et al. 2002; Gusarov 2003a). Native.

##### Comments.

This species may be distinguished from all eastern *Tinotus* but *Tinotus caviceps* based on the combination of reddish body and elytra with short, bristle-like setae that are directed obliquely laterad (Klimaszewski et al. 2002). The aedeagi and spermathecae of *Tinotus trisectus* and *Tinotus caviceps* are extremely similar and there was previously some doubt whether these two species were distinct due to the limited available material of *Tinotus trisectus* (Klimaszewski et al. 2002). Gusarov (2003a) also followed this concept of the two species, corrected a synonymy and provided additional records for *Tinotus trisectus*. After examination of Ontario specimens of *Tinotus caviceps* and *Tinotus trisectus* we provide further evidence to maintain the status of these species based on the following consistent and unambiguous differences: internal sac of aedeagus of *Tinotus caviceps* with lower sclerite hooked ventrally in lateral view, not hooked in *Tinotus trisectus*; in both sexes, antennomere III of *Tinotus caviceps* strongly flattened and broadened in lateral view, cylindrical in *Tinotus trisectus*; elytral suture of *Tinotus caviceps* slightly but distinctly shorter than length of pronotum at midline, approximately the same length or longer in *Tinotus trisectus*.

*Tinotus trisectus* appears to prefer open habitats including woodland edges, agricultural fields and oak savannah. Previously, nothing was known about its habitat associations. This species is probably broadly distributed across North America, reaching its northern limit in southern Canada.

### Tribe Hoplandriini Casey, 1910

#### Hoplandria klimaszewskii

Génier, 1989 New Ontario Record

http://species-id.net/wiki/Hoplandria_klimaszewskii

Fig. 6 Map 6 genitalia in Génier (1989)

##### Material examined.

CANADA: ON:*Essex Co*., Windsor, Ojibway Prairie, unburnt forest, yellow pans, 19 to 22.vi.2001, S.M. Paiero, 1 (DEBU); *Hald.-Norfolk Reg*., Cronmiller Prop., ~6km W St. Williams, 42°40'21"N, 80°29'26"W, forest, at lights, 20.vii.2011, Brunke & Paiero, 1 (DEBU), Turkey Point Prov. Park, site 1, 42°41'48"N, 80°19'48"W, forest, malaise pans, 15.vi to 5.vii.2011, Brunke & Paiero, 1 (DEBU).

##### Distribution.

Canada: ON, QC; USA: AR, DC, FL, IL, MD, NJ, NY, NC, VA, WV (Génier 1989). Native.

#### Hoplandria laevicollis

(Notman, 1920) New Ontario Record

http://species-id.net/wiki/Hoplandria_laevicollis

**Figures 7–12.**
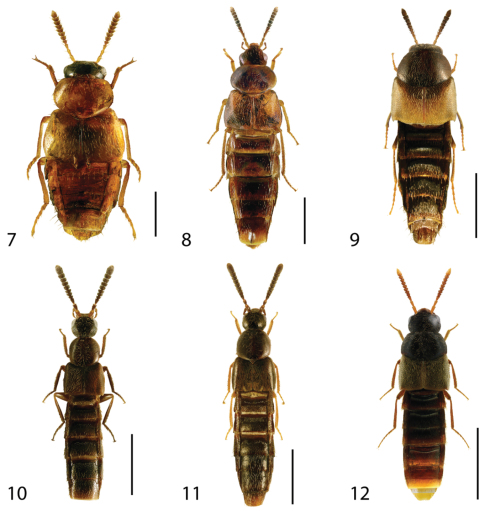
Dorsal habitus of: **7** *Hoplandria laevicollis* (Notman) **8** *Hoplandria laeviventris* Casey **9** *Platandria carolinae* Gyllenhal **10** *Amarochara brevios* Assing **11** *Amarochara fenyesi* Blatchley **12** *Crataraea suturalis* (Mannerheim). Scale 1mm.

Fig. 7 Map 7 genitalia in Génier (1989)

##### Material examined.

CANADA: ON:*Algoma Distr*., Hilton Beach, hardwood forest and field, malaise, 14 to 17.vii.1987, F.W. & J.H. Swann, 1 (DEBU).

##### Distribution.

Canada: ON, QC; USA: DC, FL, GA, LA, NC, NJ, NY, VA (Génier 1989). Native.

#### Hoplandria laeviventris

Casey, 1910 New Canadian Record

http://species-id.net/wiki/Hoplandria_laeviventris

Fig. 8 Map 8 genitalia in Génier (1989)

##### Material examined.

CANADA: ON: *Chatham-Kent Co*., Rondeau Prov. Park, int. tr. 4 (=intercept trap 4), in a white pine stand, 2.vi to 6.vi.1985, L. LeSage & A. Smetana, 1 (CNC); *Elgin Co*., Orwell, 15.vi.1978, J.M. Cumming, 1 (DEBU).

##### Distribution.

Canada: ON; USA: AL, AR, CT, DC, GA, IL, IN, KY, LA, MA, MD, NJ, NY, NC, OH, PA, TN, TX, VA, WV (Génier 1989). Native.

#### Platandria carolinae

Casey, 1910 New Canadian Record

http://species-id.net/wiki/Platandria_carolinae

**Maps 9–12.**
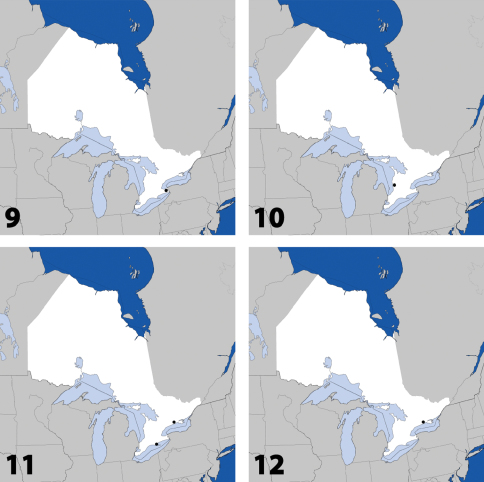
Distribution in Ontario of: **9.** *Platandria carolinae* Gyllenhal **10.** *Amarochara brevios* Assing **11** *Amarochara fenyesi* Blatchley **12** *Crataraea suturalis* (Mannerheim).

Fig. 9 Map 9 genitalia in Génier and Klimaszewski (1986)

##### Material examined.

CANADA: ON:*Lincoln Co*., Short Hills, Wildlife Pres., 1 mi E of N. Pelham, flowers of ‘Cornis florida’ L., 5.vi.1973, H. Frania, 2 (CNC).

##### Distribution.

Canada: ON; USA: DC, GA, IL, IN, IA, KA, LA, NE, NJ, NC, PA, TN, VA (Génier and Klimaszewski 1986). Native.

##### Comments.

This is the only eastern species of *Platandria* (Génier and Klimaszewski 1986) and the first record of this species for Canada. Hanley (2003) reported *Platandria* from Canada (Ontario) for the first time in a summary of its general distribution but without locality information. Little is known about the biology of *Platandria* except that they are associated with the flowers of various shrubs (Ashe in Newton et al. 2000), despite an older account of an association with fungi (Blatchley 1910). The above specimens were collected in the flowers of Flowering Dogwood, a species confined in Canada to the Carolinian region of southern Ontario. As far as known, *Platandria carolinae* is similarly distributed in Canada, possibly indicating a staphylinid-plant association though this species was not among the assemblage of Coleoptera found on Flowering Dogwood by Rhoades et al. (2011) in Tennessee.

### Tribe Oxypodini Thomson, 1859

#### Amarochara brevios

Assing, 2002 New Canadian Record

http://species-id.net/wiki/Amarochara_brevios

Fig. 10 Map 10 genitalia in Assing (2002)

##### Material examined.

CANADA: ON:*Huron Co*.,Auburn, hedgerow, pitfall trap, 26.v.2010, A. Brunke, 1 (DEBU), Auburn, soybean field, 23.vi.2010, 1 (DEBU), same data except: 7.vii.2010, 1 (DEBU), 4.viii.2010, 3 (DEBU).

##### Distribution.

Canada: ON; USA: KS (Assing 2002). Native.

##### Comments.

This species is distinguished from other Nearctic *Amarochara* based on the extremely dense punctation of the abdominal tergites, weak microsculpture of the forebody and shape of the median lobe of the aedeagus in lateral view.

*Amarochara brevios* was previously known only from the holotype collected in Kansas via flight intercept trap. Here, we report this species from Ontario, Canada based on numerous specimens collected using pitfall and raised pan traps in soybean fields (only 6 specimens kept as vouchers). While *Amarochara inquilina* (Casey) and *Amarochara formicina* Assing are associated with mound-building *Formica* ants, other species of the genus appear to be general inhabitants of decaying litter and only occasionally inhabit ant nests (Assing 2002). Currently nothing is known about the habitat preferences of other Nearctic *Amarochara*. Based on recent collections of *Amarochara* in Canada (Assing 2007, Webster et al. 2009; this study), species of this genus are poorly collected but widespread across eastern North America and all four Nearctic species are now reported from Canada (see below).

#### Amarochara fenyesi

Blatchley, 1910 New Canadian Record

http://species-id.net/wiki/Amarochara_fenyesi

Fig. 11 Map 11 genitalia in Assing (2002)

##### Material examined.

CANADA: ON:*Hald.-Norfolk Reg*.,Cronmiller Prop., ~6km W St. Williams, 42°40'18"N, 80°29'24"W, low forest, malaise pans, 5 to 17.viii.2011, Brunke & Paiero, 1 (DEBU), same data except: 42°40'20"N, 80°29'29"W, ridge forest, malaise pans, 17.viii to 1.ix.2011, 1 (DEBU), 42°40'20"N, 80°29'29"W, ridge forest, malaise pans, 1.ix to 20.ix.2011, Brunke & Paiero, 1 (DEBU; *Northumberland Co*.,Peter's Woods Prov. Nat. Res., 44°7'26"N, 78°2'31"W, forest, malaise pans, backwoods, 19.v to 1.vi.2011, Brunke & Paiero, 1 (DEBU), same data except: front woods, 16 to 27.vi.2011, 1 (DEBU), 12 to 26.vii.2011, 1 (DEBU), 12 to 26.viii.2011, 2 (DEBU).

##### Distribution.

Canada: ON; USA: GA, IN, KS (Assing 2002). Native.

##### Comments.

This species can by distinguished from other Nearctic *Amarochara* by the following combination of characters: head and pronotum with weak microsculpture; first segment of metatarsus about as long as second to fourth segments combined; punctation of abdominal tergites sparse (Assing 2002, 2007). The shape of the median lobe of the aedeagus is also distinctive in lateral view.

All specimens of this species with collection data were collected in forested reserves using flight intercept traps (Assing 2002, this study) but nothing further is known about its biology.

#### Crataraea suturalis

(Mannerheim, 1830) New Ontario Record

http://species-id.net/wiki/Crataraea_suturalis

Fig. 12 Map 12 genitalia in Klimaszewski et al. (2007a)

##### Material examined.

CANADA: ON:*Northumberland Co*., Barr property, 7 km NE Centreton, site 1, 44°7'40"N, 77°58'57"W, savannah, malaise pans, 16 to 27.vi.2011, Brunke & Paiero, 1 (DEBU).

##### Distribution.

Canada: BC, SK, ON, NB, NS, NL; USA: CA, IA, IL, IN, MA, MO, PA, SC, VA, VT; widespread in Palaearctic (Moore and Legner 1975; Seevers 1978; Downie and Arnett 1996; Smetana 2004; Klimaszewski and Majka 2007a; Webster et al. 2009; Klimaszewski et al. 2011). Adventive in Canada.

#### Dexiogyia angustiventris

(Casey, 1894)

http://species-id.net/wiki/Dexiogyia_angustiventris

**Figures 13–18.**
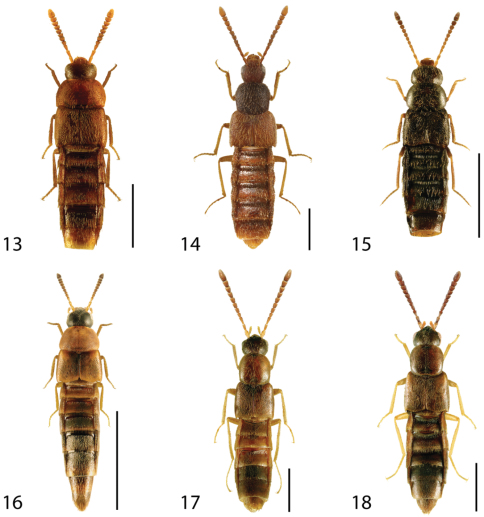
Dorsal habitus of: **13** *Dexiogyia angustiventris* (Casey) **14** *Ilyobates bennetti* Donisthorpe **15** *Ocyusa canadensis* Lohse **16** *Oxypoda rubescans* Casey **17** *Parocyusa americana* (Casey) **18** *Parocyusa fuliginosa* (Casey). Scale 1mm.

**Maps 13–16.**
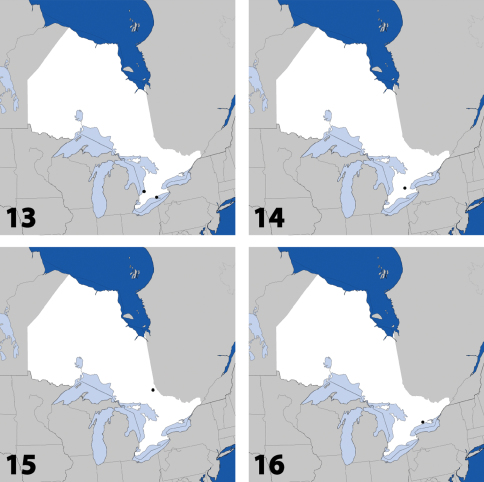
Distribution in Ontario of: **13** *Dexiogyia angustiventris* (Casey) **14** *Ilyobates bennetti* Donisthorpe **15** *Ocyusa canadensis* Lohse **16** *Oxypoda rubescens* Casey.

Figs 13 83–89 Map 13

Thiasophila angustiventris Casey, 1894: 303; 1911: 16. Lectotype (male). Iowa; *angustiventris*-3, paratype NMNH 39754; Casey bequest 1925; male; our lectotype designation label, present designation (NMNH). Paralectotypes: Iowa; Type NMNH 39754; Casey bequest 192 (NMNH) 1 female [dissected, missing spermatheca]; Iowa; *angustiventris*-2, paratype NMNH 39754; Casey bequest 1925 (NMNH) 1 female [undissected].
Thiasophila asperata Casey, 1894: 303 syn. n.. Lectotype (female). California; *Thiasophila asperata* Casey; Type NMNH 39757; Casey bequest 1925; our lectotype designation label, present designation (NMNH) [dissected].
Ischnoglossa abscissa Casey, 1911: 16 syn. n.. Holotype (male). Rhode Island (Boston Neck in orig. descr.); male; *Ischnoglossa abscissa* Casey; Type NMNH 39753, Casey bequest 1925 (NMNH) [dissected].
Ischnoglossa tenuicauda Casey, 1911: 17 syn. n.. Holotype (male). Florida; male; *Ischnoglossa tenuicauda* Casey; Type NMNH 39755, Casey bequest 1925 (NMNH) [dissected].
Ischnoglossa intenta Casey, 1911: 17 syn. n.. Lectotype (male). Iowa, Iowa City, Wickham; *intenta* Casey; Type NMNH 39756, Casey bequest 1925; our lectotype designation label, present designation (NMNH), [dissected].
Ischnoglossa alticola Casey, 1911: 18 syn. n.. Holotype (female). California (Truckee in orig. descr.);*alticola* Casey;Type NMNH 39758; Casey bequest 1925 (NMNH) [dissected, missing spermatheca].
Dexiogyia angustiventris (Casey); Seevers 1978: 68 (as ‘anguliventris’). New Canadian Record

##### Material examined.

(Type material – see above). CANADA: ON:*Hald.-Norfolk Reg*., Cronmiller prop., 6 km W St Williams, site 2, 42°40'18"N, 80°29'24"W, forest, malaise pans, 17.v to 31.v.2011, A. Brunke & S.M. Paiero, 1 (DEBU) same data except: 31.v. to 15.vi; Cronmiller prop., 6 km W St Williams, 42°40'21"N, 80°29'26"W, forest, 5.vii.2011, A. Brunke, 1 (DEBU); *Lambton Co*.,Pinery Prov. Pk., under white pine bark, 17.iv.2010, A. Brunke, 2 (DEBU).

##### Distribution.

Canada: ON; USA: CA, FL, IA, RI. Native.

##### Comments.

All North American species of *Dexiogyia* were described by Casey (1894, 1911) (as *Thiasophila* and *Ischnoglossa*) and differentiated based on slight differences in body proportions, punctation, pubescence and color. An examination of the relevant types revealed no differences between them in their aedeagi or spermathecae and slight differences in external morphology, which were attributed to intraspecific variation. Therefore, *Dexiogyia angustiventris* was selected as the valid name for this species based on its appearance before *Dexiogyia asperata* (Casey) in Casey (1894), and *Dexiogyia asperata* (Casey) syn. n., *Dexiogyia abscissa* (Casey) syn. n., *Dexiogyia tenuicauda* (Casey) syn. n., *Dexiogyia intenta* (Casey) syn. n.and *Dexiogyia alticola* (Casey) syn. n.are here placed in synonymy with *Dexiogyia angustiventris* (Casey). To provide nomenclatural stability we have selected and designated lectotypes for *Thiasophila angustiventris* Casey, *Thiasophila asperata* Casey and *Ischnoglossa intenta* Casey. Additionally, one non-type specimen (Iowa, male) of *Dexiogyia angustiventris* and five non-type specimens in Casey’s collection (NMNH) of *Dexiogyia alticola* (California, Siskiou Co., 3 females, 1 male, 1 sex?) were examined.

Seevers (1978) noted that the European species *Dexiogyia corticina* (Erichson) was probably distinct from *Dexiogyia angustiventris* based on the longer and shaper teeth on male tergite 8 in the latter species. After examination of dissected specimens of *Dexiogyia corticina* from Leipzig, Saxonia, Germany (ZMB), we consider both as valid but extremely similar species. *Dexiogyia corticina* may be distinguished from the Nearctic *Dexiogyia angustiventris* based on the tubus of the median lobe with a ventral swelling in lateral view (straight in *Dexiogyia angustiventris* (Fig. 84) and the shorter, obtuse teeth of male tergite 8 (Fig. 85).

*Dexiogyia* has been associated with subcortical microhabitats, especially those of pine and in the ‘burrows of wood-boring beetles’ (Seevers 1978). This is the first record of the genus from Canada and due to its association with pine (Seevers 1978), we suspect this species to be transcontinental in Canada.

#### Ilyobates bennetti

Donisthorpe, 1914 New Ontario Record

http://species-id.net/wiki/Ilyobates_bennetti

Fig. 14 Map 14 genitalia in Assing (1999)

##### Material examined.

CANADA: ON:*Waterloo Reg*.,Blair, hedgerow, pitfall trap, 5.v.2009, A. Brunke, 5 (DEBU), same data except: 19.v.2009, 1 (DEBU).

##### Distribution.

Canada: ON, QC, NB, NS; widespread in Palaearctic (Assing 1999; Smetana 2004; Majka and Klimaszewski 2008b; Webster et al. 2009). Adventive in Canada.

#### Ocyusa canadensis

Lohse, 1990 New Ontario Record

http://species-id.net/wiki/Ocyusa_canadensis

Fig. 15 Map 15 genitalia in Lohse et al. (1990)

##### Material examined.

CANADA: ON: *Timiskaming Distr*., 52 mi S of Armstrong, 27.vi.1973, R. Parry & J.M. Campbell, 7 (CNC).

##### Distribution.

Canada: YT, ON; USA: AK (Lohse et al. 1990). Native.

##### Comments.

The specimens from boreal Ontario represent the first record of this species in eastern North America and suggest a transboreal

##### distribution.

#### Oxypoda rubescans

Casey, 1911 New Canadian Record

http://species-id.net/wiki/Oxypoda_rubescans

Figs 16 90 Map 16

Oxypoda rubescans Casey 1911: 26–27. Lectotype (male). USA: New York, [Catskill Mts.]; *rubescans* Casey, Type USNM 39802; Casey bequest 1925; our lectotype designation label, present designation (NMNH) [dissected]

##### Material examined.

(Type material – see above). CANADA: ON:*Northumberland Co*., Barr prop., 7 km NE Centreton, site 2, 44°7'48"N, 77°59'3"W, field, malaise pans, 19.v to 1.vi.2011, Brunke & Paiero, 1 (DEBU).

##### Distribution.

Canada: ON; USA: NY. Native.

##### Comments.

This is the first collection of *Oxypoda rubescans* since its description based on a male specimen collected in the Catskill Mountains of New York (Casey 1911). The aedeagus of this species is illustrated for the first time (Fig. 90). This species is similar in habitus to *Oxypoda hiemalis* Casey but is immediately differentiated by the elytra, which are longer than the pronotum at suture. *Oxypoda rubescans* may be easily recognized by the distinctively shaped median lobe of the aedeagus in lateral view (Fig. 90). To promote nomenclatural stability, we designate a lectotype for this species here.

#### Parocyusa americana

(Casey, 1906) New Canadian Record

http://species-id.net/wiki/Parocyusa_americana

**Maps 17–20.**
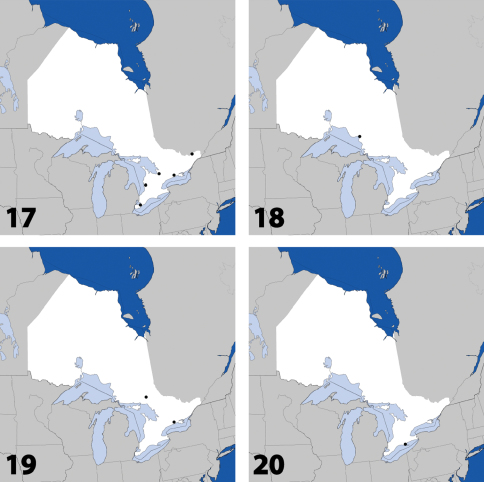
Distribution in Ontario of: **17** *Parocyusa americana* (Casey) **18** *Parocyusa fuliginosa* (Casey) **19** *Brachyusa helenae* (Casey) **20** *Gnypeta helenae* Casey.

Figs 17 91–93 Map 17

Chilopora americana Casey 1906: 306. Lectotype (female):USA, New York, Peekskill; 555, Type USNM 39734; *Chilopora americana* Casey; our lectotype designation label, present designation (NMNH) [dissected].
Tetralaucopora americana (Casey); Moore and Legner 1975: 493
Parocyusa americana (Casey); Ashe in Newton et al. 2000: 362

##### Material examined.

(Type material – see above). CANADA: ON:*Chatham-Kent Co*.,Tilbury, pitfall trap, 23.vi.1994, T. Savinski, 1 (DEBU); *Huron Co*.,Auburn, hedgerow, pitfall, 27.x.2010, A. Brunke, 1 (DEBU); *Northumberland Co*.,Peter's Woods PNR, 44°7'27"N, 78°2'21"W, dry streambed, under rock, 12.ix.2011, Brunke & Paiero, 1 (DEBU); *Ottawa Division*, Mer Bleue, 20.ix.1980, leg. R. Baranowski, 1 (MZLU); *Simcoe Co*.,Midhurst, Finlay Mills Rd., Willow Creek, 44°26'24"N, 79°43'48"W, splashing sandy bank, 13.vi.2010, A. & K. Brunke, 1 (DEBU).

##### Distribution.

Canada: ON; USA: NY. Native

##### Comments.

This is the first record of *Parocyusa americana* since its description based on a female specimen collected from Peekskill, New York (Casey 1906). This species is easily recognized to genus by its habitus and the only other known Nearctic species (*Parocyusa fuliginosa* (Casey)) is darker, with a slightly shorter and more densely punctate pronotum, and has quadrate to slightly transverse antennomeres 8–10 (see Fig. 28 in Klimaszewski et al. 2011). To promote nomenclatural stability, we designate a lectotype for *Parocyusa americana* here.

Specimens of *Parocyusa americana* were found on a stream bank and in a dry streambed under a rock. We expect *Parocyusa americana* to occur broadly over northeastern North America in habitats near running water.

#### Parocyusa fuliginosa

(Casey, 1906) New Ontario Record

http://species-id.net/wiki/Parocyusa_fuliginosa

Figs 18 94–101 Map 18 genitalia in Klimaszewski et al. (2011)

Chilopora fuliginosa Casey 1906: 307. Lectotype (female):USA, North Carolina; [Asheville in orig. description]; Type USNM 39735; *fuliginosa* Casey; our lectotype designation label, present designation (NMNH). Paralectotype (male):USA, Pennsylvania, Phila Neck, 1–14; *fuliginosa*-2, Paratype USNM 39735 (NMNH).
Tetralaucopora fuliginosa (Casey); Moore and Legner 1975: 493
Parocyusa fuliginosa (Casey); Ashe in Newton et al. 2000: 362

##### Material examined.

CANADA: ON: *Algoma Distr*., Michipicoten River, south of Wawa, 5.ix.1980, leg. R. Baranowski, 1 (MZLU).

**Figures 19–24.**
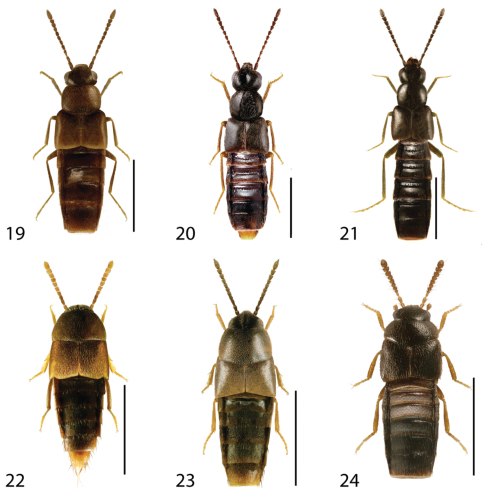
Dorsal habitus of: **19** *Brachyusa helenae* (Casey) **20** *Gnypeta helenae* Casey **21** *Gnypeta nigrella* (LeConte) **22** *Myllaena cuneata* Notman **23** *Myllaena potawatomi* Klimaszewski **24** *Agaricomorpha websteri* Klimaszewski & Brunke sp. n.Scale 1mm.

##### Distribution.

Canada: ON, NL; USA: MA, NC, PA (Seevers 1978). Native.

##### Comments.

This species was recorded from Canada for the first time by Klimaszewski et al. (2011) based on a specimen collected in Labrador, Newfoundland. The identification of this specimen was based on information provided in Seevers (1978) because the type material could not be located in the NMNH. This material was recently found and we here confirm the identity of the Newfoundland specimen as *Parocyusa fuliginosa*, newly record it from Ontario and designate a lectotype to promote nomenclatural stability. *Parocyusa fuliginosa* has been collected in much the same way as *Parocyusa americana* and we expect both species to occur broadly in eastern North America in habitats near running water.

### Tribe Tachyusini Thomson 1859

#### Brachyusa helenae

(Casey, 1911) New Ontario Record

http://species-id.net/wiki/Brachyusa_helenae

Fig. 19 Map 19 genitalia in Klimaszewski et al. (2011)

##### Material examined.

CANADA: ON:*Northumberland Co*., Peter’s Woods PNR, 44°7'27"N, 78°2'21"W, forest, 12.vii.2011, A. Brunke, 1 (DEBU); *Greater Sudbury Div*., Wahnapitae, 22.viii.1980, leg. R. Baronowski, 1 (MZLU).

##### Distribution.

Canada: YT, NT, ON, NL; USA: AK, MT (Campbell and Davies 1991; Klimaszewski et al. 2011; Klimaszewski et al. 2012). Native.

#### Gnypeta helenae

Casey, 1906 New Ontario Record

http://species-id.net/wiki/Gnypeta_helenae

Fig. 20 Map 20 genitalia in Klimaszewski et al. (2008b)

##### Material examined.

CANADA: ON:*Hald.-Norfolk Reg*., Cronmiller prop., ~6km W St. Williams, 42°40'20"N, 80°29'29"W, eutrophic pond edge, 17.viii.2011, A. Brunke, 1 (DEBU), same data except S.M. Paiero, 1 (DEBU).

##### Distribution.

Canada: BC, AB, ON; USA: AZ, MT, NM, OR (Moore and Legner 1975; Klimaszewski et al. 2008). Native.

##### Comments.

This is the first record of this species from eastern North America. *Gnypeta helenae* is indistinguishable externally from *Gnypeta canadensis* Klimaszewski, which was described based on characters of the male and female genitalia (Klimaszewski et al. 2008b). The authors noted that a wide geographic range of specimens was not available for examination and further study may necessitate re-examination of these species concepts. Study of recent material of both species from the same locality in Haldimand-Norfolk Region, Ontario, Canada confirmed that *Gnypeta helenae* and *Gnypeta canadensis* are indeed separate but cryptic species. Specimens of *Gnypeta helenae* with label data have been collected on the banks of rivers and lakes and from a eutrophic pond edge (Ontario specimen), while those of *Gnypeta canadensis* were collected in forested wetland habitats and some of these were hand collected from moss on deadwood (Ontario material). Further collecting in wet microhabitats may reveal ecological differences in these two species.

#### Gnypeta nigrella

(LeConte, 1863) New Ontario Record

http://species-id.net/wiki/Gnypeta_nigrella

**Maps 21–24.**
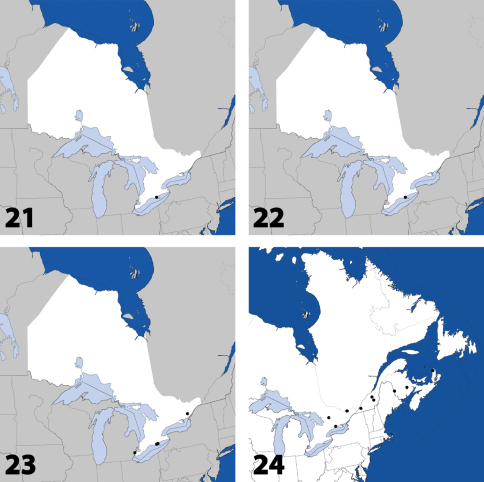
Distribution in Ontario of: **21** *Gnypeta nigrella* (LeConte) **22** *Myllaena cuneata* Notman **23** *Myllaena potawatomi* Klimaszewski. World distribution of: **24** *Agaricomorpha websteri* Klimaszewski & Brunke sp. n.

Fig. 21 Map 21 genitalia in Klimaszewski et al. (2008b)

##### Material examined.

CANADA: ON:*Hald.-Norfolk Reg*., Cronmiller Prop., ~6km W St. Williams, 42°40'20"N, 80°29'29"W, eutrophic pond, treading edge, 4.viii.2011, Brunke & Paiero, 1 (DEBU), same data except: 17.viii.2011, A. Brunke, 1 (DEBU).

##### Distribution.

Canada: ON, NB, NL;USA: MA, MD, PA, VT (Moore and Legner 1975; Klimaszewski et al. 2008b; Klimaszewski et al. 2011). Native.

### Tribe Myllaenini Ganglbauer 1895

#### Myllaena cuneata

Notman, 1920 New Ontario Record

http://species-id.net/wiki/Myllaena_cuneata

Fig. 22 Map 22 genitalia in Klimaszewski (1982b)

##### Material examined.

CANADA: ON:*Hald.-Norfolk Reg*., Cronmiller Prop., ~6km W St. Williams, 42°40'21"N, 80°29'26"W, forest, at lights, 20.vii.2011, Brunke & Paiero, 1 (DEBU), Cronmiller Prop., ~6km W St. Williams, 42°40'21"N, 80°29'26"W, forest, Berlese leaf and log litter, 20.ix.2011, A. Brunke, 1 (DEBU).

##### Distribution.

Canada: ON, NS; USA: AR, FL, GA, IL, LA, MA, MD, NH, OK, TN, VA (Klimaszewski 1982b; Klimaszewski and Génier 1986; Klimaszewski and Frank 1992b; Majka and Klimaszewski 2010). Native.

#### Myllaena potawatomi

Klimaszewski, 1982 New Canadian Record

http://species-id.net/wiki/Myllaena_potawatomi

Fig. 23 Map 23 genitalia in Klimaszewski (1982b)

##### Material examined.

CANADA: ON:*Essex Co*., Ojibway Prairie Prov. Nat. Reserve, pond edge, 23.vii.2011, S.M. Paiero, 1 (DEBU); *Hald.-Norfolk Reg*., Cronmiller Prop., ~6km W St. Williams, 42°40'21"N, 80°29'26"W, treading edge, eutrophic pond, 4.viii.2011, Brunke & Paiero, 2 (DEBU) same data except: 12.viii.2011, S.M. Paiero, 1 (DEBU), Turkey Point Prov. Park, marsh nr. fish hatchery, treading vegetation, 20.vii.2011, A. Brunke, 3 (DEBU); *Leeds and Grenville Co*., Chaffey’s Locks, Queens Univ. Biol. Station, 44.56–76.32, in decaying veg. on lake shore, 16 to 17.viii.2010, A. Brunke, 1 (DEBU).

##### Distribution.

Canada: ON; USA: AZ, AL, CA, FL, GA, IL, IN, MA, OK, TX, VA, WI;Mexico, Haiti, Jamaica (Klimaszewski 1982b; Klimaszewski and Frank 1992b). Native.

### Tribe Homalotini Heer 1839

#### Agaricomorpha websteri

Klimaszewski & Brunke, sp. n.

urn:lsid:zoobank.org:act:684EAFCD-F04D-4237-A3EA-140839A5C588

http://species-id.net/wiki/Agaricomorpha_websteri

Figs 24 103–105 Map 24

##### Type locality.

Canada, New Brunswick, Queens Co., Cranberry Lake P.N.A., red oak forest, 46.1125°N, 65.6075°W.

##### Type material.

Holotype(male): CANADA: NB:*Queens Co*., Cranberry Lake P.N.A., 46.1125°N, 65.6075°W, 25.vi-1.vii.2009, Red oak forest, Lindgren funnel trap, R. Webster & M-A. Giguère (LFC).

Paratypes (5 males, 2 females, 6 sex unknown): CANADA: NB: *Carleton Co*., near Belleville, 1.3 km E ict. Rt. 540 & Plymouth Rd., 46.1867°N, 67.6817°W, 7-v-2008, R. Webster coll., 1 male (RWC); NS: Cape Breton H.N.P., Lone Shieling, vii.1983, Malaise trap, R. Vockeroth, PG729861, 2 sex? (CNC); ON: *Haliburton Co*.**,** 10km SE Dorset, 45.16–78.84, vernal pool litter, previously wet, 25-ix-2009, S. Kullik, 1 male (DEBU); *Northumberland Co*., Peter's Woods PNR, back woods, 44°7'28"N, 78°2'14"W, forest, malaise pans, 19-v to 1-vi-2011, Brunke & Paiero, debu01146638, 1 female (DEBU); QC: *Communaute-Urbainé-de-l'Outaouais*,Gatineau Pk., near Hull, 28.iv.1974, A. Davies, 1 sex? (CNC); *L'Aminate*, Ste-Praxède, 6–13.vii.1999, Lindgren trap # 3, 99–3-0461, 2 sex? (LFC), Saint-Jacques-de-Leeds, 46°16'N, 71°23'W, 7.vii-9.vii.1993, Plan Vert ‘93, Lindgren trap # 1, Dispositif B, Ėrabliėre [=sugar bush], ‘1993–3-0381', Hébert & Jobin, 1 female (LFC); *Rousillon*,Ste-Catherine, Port., 29.vi.1961, 5.viii, 9.viii, 26.viii.1961, J-C. Aubé, 3 males, 1 sex? (CNC).

##### Description.

Body small, compact and oval in outline;length 1.6–1.8 mm; body dark brown with legs, maxillary palpi and 2–3 basal antennomeres yellowish-brown, or body dark brown with pronotum and elytra slightly paler, and appendages and basal part of abdomen yellowish-brown (Fig. 24); forebody with strong meshed microsculpture, punctation coarse, sparse and flatly impressed, pubescence sparse and approximately evenly distributed on forebody; head transverse and produced anteriad, eyes large and longer than postocular area, pubescence directed posteriad and obliquely mediad; ligula narrowly elongate and divided almost to base; antennae slightly incrassate, basal 3 antennomeres elongate, 4 subquadrate, 5–10 increasingly broadening apically, 11 oval and elongate; maxillary palpi with 4 articles, penultimate article expanded apically, and apical article acicular; pronotum strongly transverse, base strongly sinuate, converging apicad, disc with pubescence directed posteriad except for some setae at base directed laterad; elytra at suture distinctly longer than pronotum, pubescence directed straight posteriad; abdomen gradually but weakly tapering apicad, tergites II and III impressed basally, and with elevated punctures.

Male. Tergite VIII transverse, shallowly emarginate medially at the apical margin and with short medio-apical carinate protuberance (Fig. 104); sternite VIII broadly rounded apically (Fig. 105); median lobe of aedeagus in lateral view with large bulbus and U-shaped, narrow tubus with broad and angular swelling subapically; flagellum long, thin, everted and about 3 times as long as tubus (difficult to see in Fig. 103).

Female. Tergite VIII strongly transverse and similar to that of male but lacking median carina; sternite VIII transverse and arcuate apically; spermatheca with spherical capsule and inconspicuous short stem, in general similar to those of *Gyrophaena* and *Eumicrota*.

##### Distribution.

Known from Ontario, Quebec, New Brunswick and Nova Scotia. *Agaricomorpha websteri* is probably broadly distributed in northeastern North America, south of the boreal forest zone.

##### Bionomics.

Little is known about the natural history of this species but all specimens were collected in deciduous forests, mostly by passive, above-ground traps indicating high flight capability. Other species of the genus are found on woody and leathery polypore fungi (Ashe in Newton et al. 2000), which commonly grow on dead or dying standing trees. Interestingly, several individuals were captured by Lindgren funnel traps, which typically attract species associated with this type of coarse woody debris.

##### Etymology.

This species is dedicated to our colleague Reginald P. Webster of Charters Settlement, New Brunswick, who collected the holotype and whose material has contributed much to the knowledge of Canadian biodiversity.

##### Comments.

*Agaricomorpha websteri* is the only known species of the genus in eastern North America. This genus was erected by Ashe (1984) to accommodate *Agaricomorpha apacheana* (Seevers), which occurs in the southwestern United States and is not related to species of the Palaearctic genus *Agaricochara* Kraatz where it was originally described (Ashe 1984). The genus *Agaricomorpha* is distinctive among the North American Gyrophaenina for its divided ligula (Ashe in Newton et al. 2000) and strongly transverse pronotum with a distinctly sinuate base. Ashe (1984) listed *Agaricomorpha* ‘undescr. sp. 3’ as occurring in ‘Canada’ and *Agaricomorpha websteri* likely represents this taxon.

#### Eumicrota corruscula

(Erichson, 1839) New Ontario Record

http://species-id.net/wiki/Eumicrota_corruscula

**Figures 25–30.**
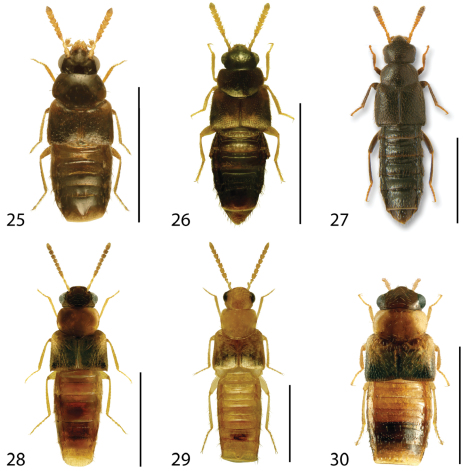
Dorsal habitus of: **25** *Eumicrota corruscula* (Erichson) **26** *Eumicrota socia* (Erichson) **27** *Euvira micmac* Klimaszewski & Majka **28** *Gyrophaena affinis* Mannerheim **29** *Gyrophaena antennalis* Casey **30** *Gyrophaena brevicollis* Seevers. Scale 1mm.

**Maps 25–28.**
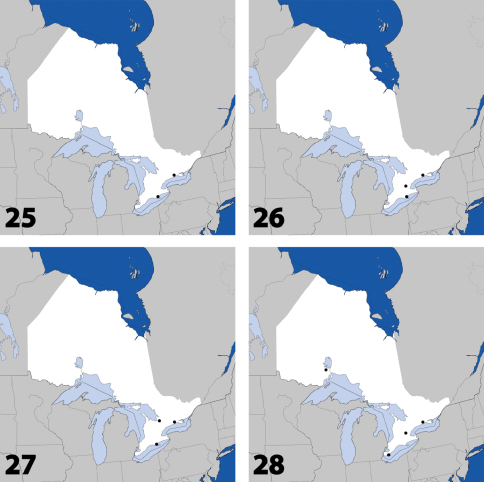
Distribution in Ontario of: **25** *Eumicrota corruscula* (Erichson) **26** *Eumicrota socia* (Erichson) **27** *Euvira micmac* Klimaszewski & Majka **28** *Gyrophaena affinis* Mannerheim.

Fig. 25 Map 25 genitalia in Klimaszewski et al. (2009)

##### Material examined.

CANADA: ON:*Hald.-Norfolk Reg*., Turkey Point Prov. Park, site 2, 42°42'28"N, 80°20'29"W, savannah, at lights, 5.viii.2011, Brunke & Paiero, 3 (DEBU); *Northumberland Co*., Peter’s Woods PNR, 44°7'27"N, 78°2'21"W, forest, on fungi, 12.viii.2011, A. Brunke, 1 (DEBU).

##### Distribution.

Canada: ON, QC, NB; USA: AL, CT*, DC, FL, GA, IL, IN, IA, KS, KY, LA, MA, MI, MO, NJ, NY, OH, PA, SC, TN, TX, VA, WI, WV (Seevers 1951; Klimaszewski et al. 2009). Native.

#### Eumicrota socia

(Erichson, 1839) New Ontario Record

http://species-id.net/wiki/Eumicrota_socia

Fig. 26 Map 26 genitalia in Klimaszewski et al. (2009)

##### Material examined.

CANADA: ON:*Hald.-Norfolk Reg*., Turkey Point Prov. Park, site 1, 42°41'48"N, 80°19'48"W, forest, on fungus, 17.viii.2011, A. Brunke, 1 (DEBU), Turkey Point Prov. Park, site 2, 42°42'28"N, 80°20'29"W, savannah, Berlese leaf and log litter w. fungus, 17.v.2011, A. Brunke, 1 (DEBU); *Northumberland Co*., Peter’s Woods Prov. Nat. Res., 44°7'27"N, 78°2'21"W, forest, Berlese leaf & log litter, 1.vi.2011, Brunke & Paiero, 1 (DEBU), Peter’s Woods Prov. Nat. Res., 44°7'26"N, 78°2'31"W, forest, malaise pans, 19.v to 1.vi.2011, Brunke & Paiero, 1 (DEBU), same data except: 16 to 27.vi.2011, 2 (DEBU); *Wellington Co*., Guelph, Arboretum, 11.ix.2007, A. Brunke, 1 (DEBU).

##### Distribution.

Canada: ON, QC, NB, NS, PE; USA: AR, DC, FL, IL, IN, KS, KY, LA, ME, MD, MI, MO, NY, NC, OH, PA, SC, TN, TX, VA, WI, WV (Seevers 1951; Klimaszewski et al. 2009; Majka and Klimaszewski 2010). Native.

#### Euvira micmac

Klimaszewski & Majka, 2007 New Ontario Record

http://species-id.net/wiki/Euvira_micmac

Fig. 27 Map 27 genitalia in Klimaszewski and Majka (2007a)

##### Material examined.

CANADA: ON:*Hald.-Norfolk Reg*.,Cronmiller Prop., ~6km W St. Williams, 42°40'20"N, 80°29'29"W, ridge forest, malaise pans, 20.ix to 12.x.2011, Brunke & Paiero, 1 (DEBU);*Northumberland Co*., Barr Property, ~ 7km NE Centreton, 44°7'44"N, 77°59'0"W, forest, sappy *Populus* wood, 12.vii.2011, A. Brunke, 1 (DEBU), Barr Property, ~ 7km NE Centreton, 44°7'48"N, 77°59'3"W, old field, malaise pans, 1 to 16.vi.2011, Brunke & Paiero, 1 (DEBU), Peter’s Woods PNR, 44°7'27"N, 78°2'21"W, forest, Berlese streamside litter, 19.v.2011, A. Brunke, 1 (DEBU); *Simcoe Co*., Midhurst, forest nr. Neretva St., 44°26'22"N, 79°42'40"W, leaf litter, 10.x.2010, A. Brunke, 2 (DEBU).

##### Distribution.

Canada: ON, NB, NS; USA: OH, MI (Klimaszewski and Majka 2007a; Webster et al. 2009). Native.

##### Comments.

This species has previously been associated with Red Oak (*Quercus rubra* L.) and some specimens have been collected inside spherical Red Oak galls (Klimaszewski and Majka 2007a). All Ontario specimens were collected in forests containing red oak or in open habitat with several small, Red Oaks. Red Oaks at the Barr property in Northumberland County possessed spherical galls but these were noticed late in the season and did not contain rove beetles when checked. *Euvira micmac* has also been collected from litter near water and from under sappy *Populus* bark (Webster et al. 2009, this study), and the association with red oak may be indirect, possibly involving a fungal food source that prefers oak tissue or the microclimate provided by oak galls.

#### Gyrophaena affinis

Mannerheim, 1830 New Ontario Record

http://species-id.net/wiki/Gyrophaena_affinis

Fig. 28 Map 28 genitalia in Klimaszewski et al. (2009)

##### Material examined.

CANADA: ON:*Essex Co*., Point Pelee, 24.vi.1925, G.S. Walley, 1 (CNC); *Northumberland Co*., Peter’s Woods PNR, 44°7'27"N, 78°2'21"W, forest, on fungus, 12.viii.2011, S.M. Paiero, 1 (DEBU); *Thunder Bay Distr*., Black Sturgeon Lake, 1 to 5.viii.1956, Lindberg, 7 (CNC); *Wellington Co*., Guelph, reared from fungus, 23.viii.1990, H. Dewer, 1 (DEBU).

##### Distribution.

Canada: BC, MB, ON, QC, NB, NS, NL; USA: AZ*, DC, IL, IN, IA, KY, ME, MA, MI, MN, MO, NC, NH, NJ, NM, NY, OH*, PA, TN, WA, WI, WV (Seevers 1951; Campbell and Davies 1991; Majka and Klimaszewski 2008a; Klimaszewski et al. 2009; Klimaszewski et al. 2011). Adventive in Canada.

##### Comments.

This adventive species was accidentally listed as occurring in Ontario in Klimaszewski et al. (2007a) and was subsequently included as occurring there in other accounts of adventive Aleocharinae (Gouix and Klimaszewski 2007; Klimaszewski et al. 2010). The above data represent the first confirmed records of this species in Ontario, as early as 1925.

#### Gyrophaena antennalis

Casey, 1906 New Ontario Record

http://species-id.net/wiki/Gyrophaena_antennalis

**Maps 29–32.**
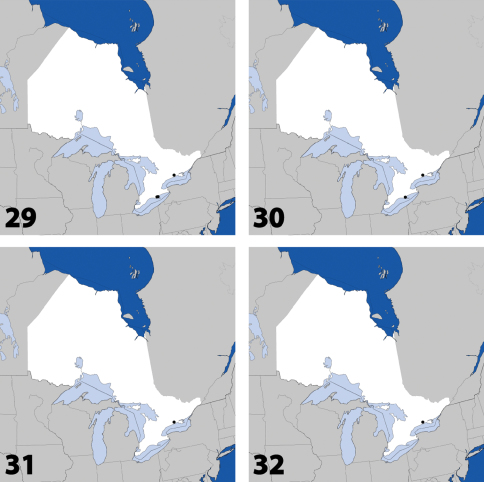
Distribution in Ontario of: **29** *Gyrophaena antennalis* Casey **30** *Gyrophaena brevicollis* Seevers **31** *Gyrophaena caseyi* Seevers **32** *Gyrophaena criddlei* Casey.

Fig. 29 Map 29 genitalia in Klimaszewski et al. (2009)

##### Material examined.

CANADA: ON:*Hald.-Norfolk Reg*., Manester Tract, 6km NNW St. Williams, 17.ix.2008, A. Brunke, 2 (DEBU), Turkey Point Prov. Park, site 1, 42°41'48"N, 80°19'48"W, forest, on fungi, 20.ix.2011, S.M. Paiero, 1 (DEBU); *Northumberland Co*., Peter’s Woods PNR, 44°7'26"N, 78°2'31"W, forest, gilled mushrooms, 12.ix.2011, A. Brunke, 1 (DEBU).

##### Distribution.

Canada: ON, NB, NS, NL; USA: MA, NC, NY, TN* (Seevers 1951; Campbell and Davies 1991; Klimaszewski et al. 2009; Majka and Klimaszewski 2010; Klimaszewski et al. 2011). Native.

##### Comments.

This species was newly recorded from Nova Scotia by Majka and Klimaszewski (2010) in a species list for the Maritime Provinces, but specimen data were accidentally omitted from the body of the text (C. Majka, *pers. comm*.). One specimen was collected from mainland Nova Scotia and was identified by one of us (JK).

#### Gyrophaena brevicollis

Seevers, 1951 New Canadian Record

http://species-id.net/wiki/Gyrophaena_brevicollis

Fig. 30 Map 30 genitalia in Seevers (1951)

##### Material examined.

CANADA: ON:*Hald.-Norfolk Reg*., Cronmiller prop., 6km W St. Williams, 42°40'21"N, 80°29'26"W, forest, 17.viii.2011, A. Brunke, 1 (DEBU), same data except: 42°40'20"N, 80°29'29"W, forest, site 1, malaise pans, 20.ix to 12.x.2011, Brunke & Paiero, 1 (DEBU); *Northumberland Co*., Peter’s Woods PNR, 44°7'27"N, 78°2'21"W, forest, gilled mushrooms, 12.ix.2011, A. Brunke, 1 (DEBU).

##### Distribution.

Canada: ON; USA: IN, IL, MS, NC (Seevers 1951). Native.

#### Gyrophaena caseyi

Seevers, 1951 New Ontario Record

http://species-id.net/wiki/Gyrophaena_caseyi

**Figures 31–36.**
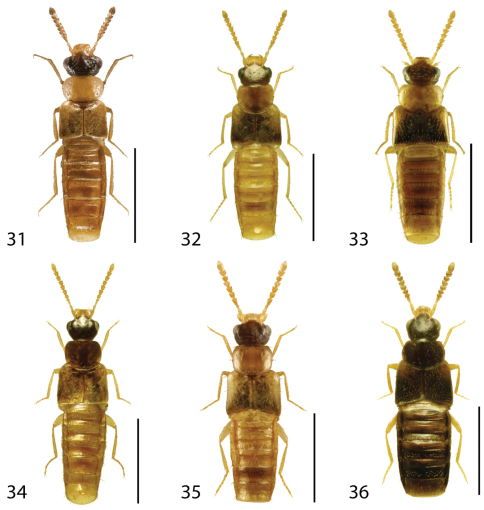
Dorsal habitus of: **31** *Gyrophaena caseyi* Seevers **32** *Gyrophaena criddlei* Casey **33** *Gyrophaena dybasi* Seevers **34** *Gyrophaena fuscicollis* Casey **35** *Gyrophaena gilvicollis* Casey **36** *Gyrophaena meduxnekeagensis* Klimaszewski and Webster. Scale 1mm.

Figs 31 106–108 Map 31

##### Material Examination.

CANADA: ON:*Northumberland Co*., Peter’s Woods PNR, 44°7'27"N, 78°2'21"W, forest, gilled mushrooms, 12.ix.2011, A. Brunke, 1 (DEBU).

##### Distribution.

Canada: ON, QC, NB; USA: MI, NC, NY, PA (Seevers 1951; Campbell and Davies 1991; Webster et al. 2012). Native.

##### Comments.

This species was erroneously reported from New Brunswick by Klimaszewski et al. (2009) based on misidentified specimens of *Gyrophaena nanoides* Seevers. *Gyrophaena caseyi* and *Gyrophaena nanoides* are very similar externally except that the former has antennomeres 5–10 distinctly transverse (elongate to quadrate in the latter) and antennomere 9 is approximately as long as 10 (longer in *Gyrophaena nanoides*). For a habitus image of *Gyrophaena nanoides* see Klimaszewski et al. (2009) (labeled as *Gyrophaena caseyi*). The median lobe of the aedeagus in lateral view is shaped slightly differently (Fig. 106 versus Fig. 39 in Klimaszewski et al. 2009 (as *Gyrophaena caseyi*)).

#### Gyrophaena criddlei

Casey, 1911 New Ontario Record

http://species-id.net/wiki/Gyrophaena_criddlei

Fig. 32 Map 32 genitalia in Klimaszewski et al. (2009)

##### Material examined.

CANADA: ON:*Northumberland Co*., Peter’s Woods PNR, 44°7'26"N, 78°2'31"W, forest, gilled mushrooms, 12.ix.2011, A. Brunke, 2 (DEBU).

##### Distribution.

Canada: YT (tentative), MB, ON, NB, NL (Seevers 1951; Campbell and Davies 1991; Klimaszewski et al. 2009; Klimaszewski et al. 2011, Klimaszewski et al. 2012). Native.

#### Gyrophaena dybasi

Seevers, 1951 New Ontario Record

http://species-id.net/wiki/Gyrophaena_dybasi

**Maps 33–36.**
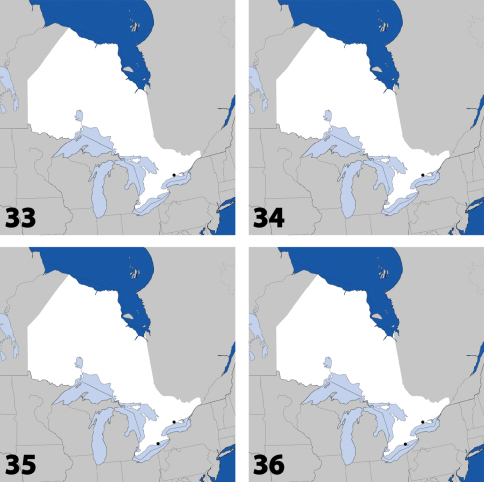
Distribution in Ontario of: **33** *Gyrophaena dybasi* Seevers **34** *Gyrophaena fuscicollis* Casey **35** *Gyrophaena gilvicollis* Casey **36** *Gyrophaena meduxnekeagensis* Klimaszewski and Webster.

Fig. 33 Map 33 genitalia in Klimaszewski et al. (2009)

##### Material examined.

CANADA: ON:*Northumberland Co*., Peter’s Woods PNR, 44°7'27"N, 78°2'21"W, forest, on fungus, 12.viii.2011, A. Brunke, 1 (DEBU).

##### Distribution.

Canada: ON, NB; USA: IL, IN, MO, NC, WI (Seevers 1951; Klimaszewski et al. 2009). Native.

#### Gyrophaena fuscicollis

Casey, 1906 New Ontario Record

http://species-id.net/wiki/Gyrophaena_fuscicollis

Fig. 34 Map 34 genitalia in Klimaszewski et al. (2009)

##### Material examined.

CANADA: ON:*Northumberland Co*., Peter’s Woods PNR, 44°7'27"N, 78°2'21"W, forest, on fungus, 12.viii.2011, S.M. Paiero, 1 (DEBU).

##### Distribution.

Canada: ON, NB; USA: DC, IL, NY, PA, WI (Seevers 1951; Klimaszewski et al. 2009). Native.

#### Gyrophaena gilvicollis

Casey, 1906 New Ontario Record

http://species-id.net/wiki/Gyrophaena_gilvicollis

Fig. 35 Map 35 genitalia in Klimaszewski et al. (2009)

##### Material examined.

CANADA: ON:*Hald.-Norfolk Reg*., Turkey Point Prov. Park, site 1, 42°41'48"N, 80°19'48"W, forest, on fungus, 20.ix.2011, S.M. Paiero, 1 (DEBU);*Northumberland Co*., Peter’s Woods PNR, 44°7'26"N, 78°2'31"W, forest, gilled mushrooms, 12.ix.2011, A. Brunke, 2 (DEBU).

##### Distribution.

Canada: ON, NB; USA: DC, IL, IN, MI, NY, PA, VA, WV (Seevers 1951; Campbell and Davies 1991; Klimaszewski et al. 2009). Native.

##### Comments.

This species was listed as questionably occurring in Ontario by Campbell and Davies (1991) based on the record from ‘Canada’ by Ashe (1984) (A. Davies *pers. comm*.). The above specimen data confirm this species’ presence in Ontario.

#### Gyrophaena meduxnekeagensis

Klimaszewski & Webster, 2009 New Ontario Record

http://species-id.net/wiki/Gyrophaena_meduxnekeagensis

Fig. 36 Map 36 genitalia in Klimaszewski et al. (2009)

##### Material examined.

CANADA: ON:*Hald.-Norfolk Reg*., Cronmiller Prop., ~6km W St. Williams, 42°40'18"N, 80°29'24"W, forest, malaise pans, 17 to 31.v.2011, Brunke & Paiero, 1 (DEBU); *Northumberland Co*., Peter’s Woods PNR, 44°7'26"N, 78°2'31"W, forest, malaise pans, 27.vi to 12.vii.2011, Brunke & Paiero, 1 (DEBU).

##### Distribution.

Canada: ON, QC, NB (Klimaszewski et al. 2009). Native.

#### Gyrophaena modesta

Casey, 1906 New Ontario Record

http://species-id.net/wiki/Gyrophaena_modesta

**Figures 37–42.**
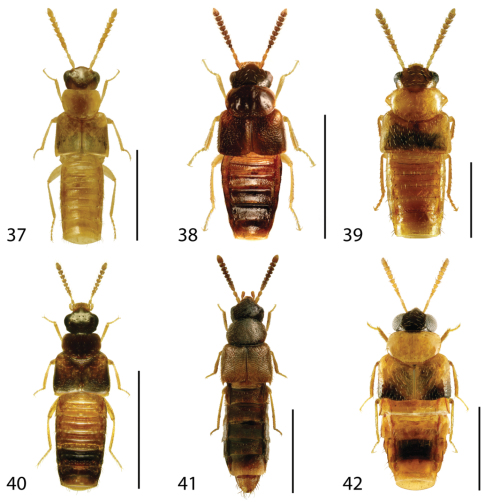
Dorsal habitus of: **37** *Gyrophaena modesta* Casey **38** *Gyrophaena neonana* Seevers **39** *Gyrophaena stroheckeri* Seevers **40** *Gyrophaena uteana* Casey **41** *Leptusa carolinensis* Pace **42** *Phanerota fasciata* (Say). Scale 1mm.

**Maps 37–40.**
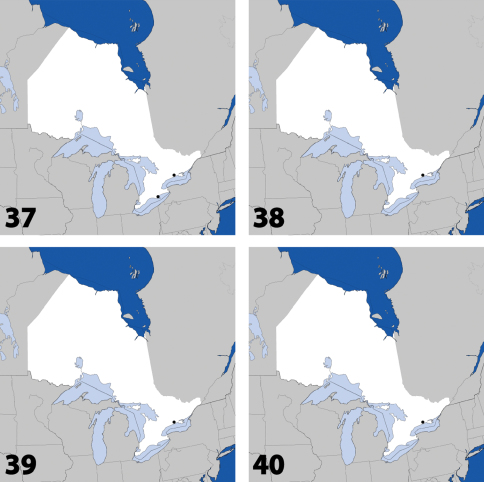
Distribution in Ontario of: **37** *Gyrophaena modesta* Casey **38** *Gyrophaena neonana* Seevers **39** *Gyrophaena stroheckeri* Seevers **40** *Gyrophaena uteana* Casey.

Fig. 37 Map 37 genitalia in Klimaszewski et al. (2009)

##### Material examined.

CANADA: ON:*Hald.-Norfolk Reg*., Turkey Point Prov. Park, site 1, 42°41'48"N, 80°19'48"W, forest, on fungus, 20.ix.2011, A. Brunke, 1 (DEBU);*Northumberland Co*., Peter’s Woods PNR, 44°7'26"N, 78°2'31"W, forest, gilled mushrooms, 12.ix.2011, A. Brunke, 2 (DEBU).

##### Distribution.

Canada: AB*, ON, NB, NS, NL; USA: IL, IN, MI, MN, NH, NY (Seevers 1951; Klimaszewski et al. 2009; Klimaszewski et al. 2011; Majka and Klimaszewski 2011). Native.

#### Gyrophaena neonana

Seevers, 1951 New Ontario Record

http://species-id.net/wiki/Gyrophaena_neonana

Fig. 38 Map 38 genitalia in Klimaszewski et al. (2008a)

##### Material examined.

CANADA: ON:*Northumberland Co*., Peter’s Woods PNR, 44°7'27"N, 78°2'21"W, forest, fungus on log, 27.vii.2011, S.M. Paiero, 3 (DEBU).

##### Distribution.

Canada: YT, ON, NB, NL; USA: IN, NC, PA, WI (Seevers 1951; Klimaszewski et al. 2008a; Klimaszewski et al. 2011; Webster et al. 2012). Native.

#### Gyrophaena stroheckeri

Seevers, 1951 New Canadian Record

http://species-id.net/wiki/Gyrophaena_stroheckeri

Fig. 39 Map 39 genitalia in Seevers (1951)

##### Material examined.

CANADA: ON:*Northumberland Co*., Peter’s Woods PNR, 44°7'27"N, 78°2'21"W, forest, fungus, 12.viii.2011, S.M. Paiero, 1 (DEBU).

##### Distribution.

Canada: ON; USA: IN, NC, WI (Seevers 1951). Native.

#### Gyrophaena uteana

Casey, 1906 New Ontario Record

http://species-id.net/wiki/Gyrophaena_uteana

Fig. 40 Map 40 genitalia in Klimaszewski et al. (2009) (*as Gy. gaudens*)

##### Material examined.

CANADA: ON:*Northumberland Co*., Barr Property, ~ 7km NE Centreton, 44°7'44"N, 77°59'0"W, field, malaise pans, 16 to 27.vi.2011, Brunke & Paiero, 1 (DEBU), Peter’s Woods PNR, 44°7'27"N, 78°2'21"W, maple-beech forest, Berlese leaf and log litter, 19.v.2011, A. Brunke, 1 (DEBU), same as previous except: 1.vi.2011, 1 (DEBU), Peter’s Woods PNR, 44°7'27"N, 78°2'21"W, forest, malaise pans, 16 to 27.vi.2011, Brunke & Paiero, 2 (DEBU).

##### Distribution.

Canada: BC, AB*, ON, QC, NB; USA: CA, CO, UT (Seevers 1951; Webster et al. 2012). Native.

#### Leptusa carolinensis

Pace, 1989 New Ontario Record

http://species-id.net/wiki/Leptusa_carolinensis

**Maps 41–44.**
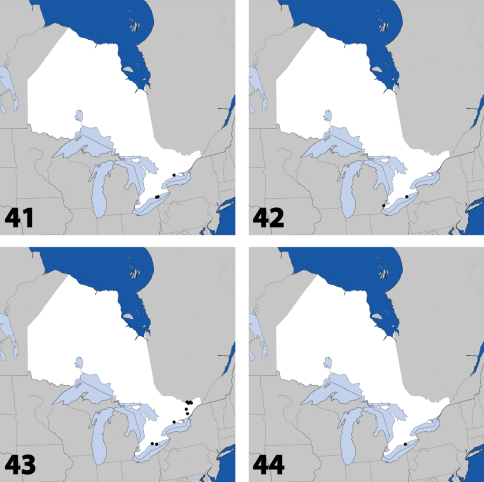
Distribution in Ontario of: **41** *Leptusa carolinensis* Pace **42** *Phanerota fasciata* (Say) **43** *Phymatura blanchardi* (Casey) **44** *Thecturota pusio* (Casey).

Fig. 41 Map 41 genitalia in Klimaszewski et al. (2004)

##### Material examined.

CANADA: ON:*Hald.-Norfolk Reg*., Cronmiller Prop., ~6km W St. Williams, 42°40'20"N, 80°29'29"W, sand ridge forest, malaise pans, 17 to 31.v.2011, Brunke & Paiero, 3 (DEBU), Turkey Point Prov. Park, wilderness area, forest, under bark, 17.v.2011, A. Brunke, 1 (DEBU);*Northumberland Co*., Peter’s Woods PNR, 44°7'27"N, 78°2'21"W, forest, under bark, large sugar maple, 6.x.2011, A. Brunke, 1 (DEBU).

##### Distribution.

Canada: ON, QC, NB, NS; USA: NC, TN (Klimaszewski et al. 2004; Webster et al. 2009; Park et al. 2010). Native.

#### Phanerota fasciata

(Say, 1834) New Canadian Record

http://species-id.net/wiki/Phanerota_fasciata

Fig. 42 Map 42 genitalia in Ashe (1986)

##### Material examined.

CANADA: ON:*Essex Co*., La Salle, Brunet Park, 29.vii.2005, S.M. Paiero, 2 (DEBU); *Hald.-Norfolk Reg*., Turkey Point Prov. Park, 42°41'48"N, 80°19'48"W, forest, gilled mushrooms, 12.x.2011, A. Brunke, 2 (DEBU).

##### Distribution.

Canada: ON; USA: AR, DC, FL, GA, IA, IL, IN, KY, KS, LA, MD, MI, MO, MS, NC, NJ, NY, OH, PA, TN, TX, VA, WI (Seevers 1951). Native.

##### Comments

**.** The genus *Phanerota* is newly recorded in Canada based on specimens collected on mushrooms in extreme southern Ontario. This genus may reach its northern distributional limit in southern Ontario, as it was not reported in a recent review of New Brunswick Gyrophaenina (Klimaszewski et al. 2009).

#### Phymatura blanchardi

(Casey, 1894) New Ontario Record

http://species-id.net/wiki/Phymatura_blanchardi

**Figures 43–48.**
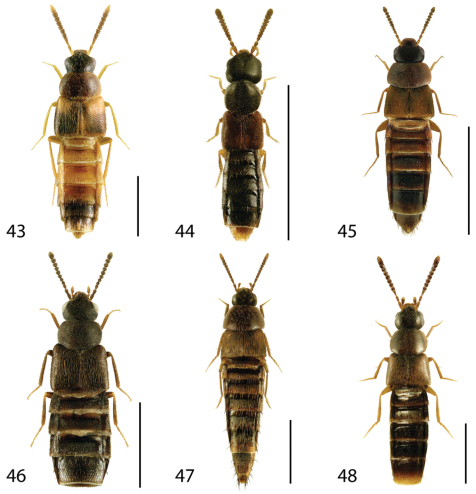
Dorsal habitus of: **43** *Phymatura blanchardi* (Casey) **44** *Thecturota pusio* (Casey) **45** *Placusa incompleta* Sjöberg **46** *Placusa vaga* Casey **47** *Acrotona smithi* (Casey) **48** *Acrotona subpygmaea* (Bernhauer). Scale 1mm.

Fig. 43 Map 43 genitalia in Ashe (1992)

##### Material examined.

CANADA: ON:*Elgin Co*., Aylmer West, malaise trap, 7 to 15.ix.1972, 1 (CNC); *Hald.-Norfolk Reg*., Cronmiller Prop., ~6km W St. Williams, 42°40'21"N, 80°29'26"W, forest, fungi, 12.viii.2011, S.M. Paiero, 1 (DEBU), same data except: 20.ix.2011, S.M. Paiero, 1 (DEBU);*Lanark Co*., Bell’s Corners, 14.x.1967, A. Smetana, 3 (CNC); *Leeds and Grenville United Co*., Chaffey’s Locks Biol. Stn., 16.x.1986, A. Smetana, 1 (CNC); *Northumberland Co*., Peter’s Woods PNR, 44°7'27"N, 78°2'21"W, forest, 6.x.2011, A. Brunke, 1 (DEBU);*Ottawa Div*., Constance Bay, x.1970, S. Peck, 1 (CNC), Leitrim, ex. *Ganoderma applanatum*, 5.x.1985, R.S. Skidmore, 1 (CNC), Ottawa, Beaulieu, 29.viii.1912, 5 (CNC), South March, 11.x.1967, J.M. Campbell & A. Smetana, 1 (CNC).

##### Distribution.

Canada: AB, ON, NB; USA: IA, IN, MO, NY (Moore and Legner 1975; Majka and Klimaszewski 2008c; Webster et al. 2009). Native.

#### Thecturota pusio

(Casey, 1894) New Canadian Record

http://species-id.net/wiki/Thecturota_pusio

Figs 44 109–115 Map 44

Oligurota pusio Casey 1894: 362
Thecturota (Oligurota) pusio (Casey); Casey 1911: 211

##### Material examined.

CANADA: ON: *Hald.-Norfolk Reg*., Turkey Point Prov. Pk., site 2, 42°42'28"N, 80°20'29"W, savannah, Berlese leaf, log and grass litter, 12.x.2011, A. Brunke, 11 (DEBU).

##### Distribution.

Canada: ON; USA: IN. Native.

##### Comments.

This is the first collection of *Thecturota pusio* since Casey’s description (1894) based on the female holotype from ‘Indiana’ and the first Canadian record of the genus. We have dissected the female holotype for comparison with the Ontario specimens and illustrate the male and female sexual characters for the first time (Figs 109–115). Live specimens of *Thecturota pusio* were extremely slow-moving and the use of a Berlese funnel likely facilitated the capture of this minute (<2mm) species.

### Tribe Placusini Mulsant & Rey, 1871

#### Placusa incompleta

Sjöberg, 1934 New Ontario Record

http://species-id.net/wiki/Placusa_incompleta

**Maps 45–48.**
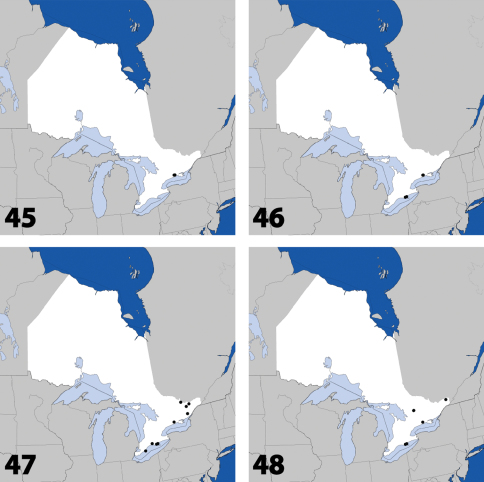
Distribution in Ontario of: **45** *Placusa incompleta* Sjöberg **46** *Placusa vaga* Casey **47** *Acrotona smithi* (Casey) **48** *Acrotona subpygmaea* (Bernhauer).

Fig. 45 Map 45 genitalia in Klimaszewski et al. (2001)

##### Material examined.

CANADA: ON:*Northumberland Co*., Peter’s Woods PNR, 44°7'26"N, 78°2'31"W, front woods, forest, malaise pans, 19.v to 1.vi.2011, Brunke & Paiero, 1 (DEBU), Barr Property, ~ 7km NE Centreton, 44°7'44"N, 77°59'0"W, forest, sappy *Populus* wood, 12.vii.2011, A. Brunke, 1 (DEBU), Barr Property, ~ 7km NE Centreton, 44°7'44"N, 77°59'0"W, 12.viii.2011, A. Brunke, 1 (DEBU).

##### Distribution.

Canada: BC, ON, QC, NB, NS, NL; USA: WA; western Palaearctic (Klimaszewski et al. 2001; Smetana 2004; Webster et al. 2009; Klimaszewski et al. 2011). Native Holarctic species or adventive in Canada.

#### Placusa vaga

Casey, 1911 New Ontario Record

http://species-id.net/wiki/Placusa_vaga

Fig. 46 Map 46 genitalia in Klimaszewski et al. (2001)

##### Material examined.

CANADA: ON:*Hald.-Norfolk Reg*., Cronmiller Prop., ~6km W St. Williams, 42°40'20"N, 80°29'29"W, forest, sand ridge, malaise, 20.vii to 5.viii.2011, Brunke & Paiero, 1 (DEBU), Turkey Point Prov. Park, 42°42'28"N, 80°20'29"W, oak savannah, malaise, 20.ix to 12.x.2011, Brunke & Paiero, 1 (DEBU); *Northumberland Co*., Barr Property, ~ 7km NE Centreton, 44°7'44"N, 77°59'0"W, forest, sappy *Populus* wood, 12.vii.2011, A. Brunke, 2 (DEBU), Barr Property, ~ 7km NE Centreton, 44°7'44"N, 77°59'0"W, 12.viii.2011, S.M. Paiero, 1 (DEBU), Peter’s Woods PNR, 44°7'27"N, 78°2'21"W, forest, 27.vii.2011, S.M. Paiero, 1 (DEBU).

##### Distribution.

Canada: YT, NT, BC, ON, QC, NB, NS; USA: CA (Klimaszewski et al. 2001; Klimaszewsk et al. 2008; Majka and Klimaszewski 2008a; Webster et al. 2009; Majka and Klimaszewski 2011). Native.

### Tribe Athetini Casey, 1910

#### Acrotona smithi

(Casey, 1910) New Canadian Record

http://species-id.net/wiki/Acrotona_smithi

Fig. 47 116–124 Map 47

Coprothassa smithi Casey, 1910: 166. Lectotype (male):USA: New York; *smithi* Casey; Type USNM 39019; Casey bequest 1925; Lectotypus male, *Coprothasa smithi* Casey, V. Gusarov des. 2000 [This designation was never published and therefore we formally designate this specimen as the lectotype]; *Acrotona smithi* (Casey), V. Gusarov 2000; our Lectotype designation label, present designation (NMNH) [dissected]. Paralectotypes (4, present designation)**:** New York; *smithi*-2, USNM 39019; Casey bequest 1925 (NMNH) 1 female. New York; *smithi*-3; Paratype USNM 39019 (NMNH) 1 female. New York; smithi-5; USNM 39019 Casey bequest 1925 (NMNH) 1 male. New York; *smithi*-6; USNM 39019 Casey bequest 1925 (NMNH) 1 male.

##### Material examined.

(Type material – see above). CANADA: ON:*Chatham-Kent Co*., Rondeau Prov. Park, end Lakeshore Rd., 1.vi.1985, A. Davies & J.M. Campbell, 1 (CNC), same data except: 5.vi.1985, sifted grass pile & leaves, 3 (CNC), Rondeau Prov. Park, deciduous forest, 19.v to 6.vii.1976, Dondale & Redner, 2 (CNC), Rondeau Prov. Park, intercept trap, on sand beach, edge oak forest, 22 to 31.vii.1985, L. LeSage & A. Woodliffe, 2 (CNC), same data except: 1 to 9.viii.1985, 1 (CNC), 9 to 17.viii.1985, 1 (CNC), Rondeau Prov. Park, intercept trap, maple-beech forest, 13 to 22.vii.1985, L. LeSage & A. Woodliffe, 1 (CNC), Rondeau Prov. Park, intercept trap, white pine stand, 1 to 9.viii.1985, L. LeSage & A. Woodliffe (2), Rondeau Prov. Park, Lakeshore Rd., 30.v.1985, A. Smetana, 2 (CNC); *Elgin Co*., Aylmer West, Malaise trap, 24 to 31.viii.1972, 1 (CNC), same data except: 15 to 22.ix.1972, 1 (CNC); *Hald.-Norfolk Reg*., Cronmiller Prop., ~6km W St. Williams, 42°40'20"N, 80°29'29"W, sand ridge, forest, malaise pans, 15.vi to 5.vii.2011, Brunke & Paiero, 1 (DEBU), same data except: 5.vii to 20.vii.2011, 1 (DEBU), 31.v to 15.vi.2011, 1 (DEBU), Cronmiller Prop., ~6km W St. Williams, 42°40'20"N, 80°29'29"W, low forest, malaise pans, 5.vii to 20.vii.2011, Brunke & Paiero, 2 (DEBU), Turkey Point Prov. Park, site 2, 42°42'28"N, 80°20'29"W, oak savannah, Lindgren funnel, 15.vi to 5.vii.2011, Brunke & Paiero, 1 (DEBU); *Lanark Co*., 7 mi W of Carleton Place, 15.v.1980, A. Smetana, 3 (CNC); *Leeds and Grenville Co*., ‘Chaffey’s Locks’ Biol. Stn., 16.x.1986, A. Smetana, 2 (CNC); *Northumberland Co*., Peter’s Woods PNR, 44°7'27"N, 78°2'21"W, forest, 12.viii.2011, A. Brunke, 1 (DEBU); *Ottawa Div*., Kanata, 25.v.1979, A. & Z. Smetana, 2 (CNC); *Renfrew Co*., Haley Sta., 15km NW Renfrew, mixed forest malaise, 2 to 30.ix.1979, S. Peck, 1 (CNC).

##### Distribution.

Canada: ON, NB; USA: NY (Webster et al. 2012). Native.

##### Comments.

*Acrotona smithi* is newly recorded in Canada based on numerous collections across Ontario. Ontario material was compared with the type series of *Acrotona smithi* from New York. This species is easily recognized amongst other northeastern *Acrotona* by the large and fusiform body (*Oxypoda*-like habitus), the distinctive shape of the aedeagus in lateral view (Figs 117–118), the broadly and shallowly emarginate female tergite VIII (Fig. 123), and, despite some variation, the general shape of the spermatheca (Figs 121–122). *Acrotona smithi* appears to be a common species inhabiting deciduous to mixed forests and semi-open habitat (e.g. oak savannah) and is probably broadly distributed across northeastern North America.

#### Acrotona subpygmaea

(Bernhauer, 1909) New Ontario Record

http://species-id.net/wiki/Acrotona_subpygmaea

Figs 48 125–131 Map 48

Atheta subpygmaea Bernhauer, 1909: 526. Lectotype (female): Massachusetts, Framingham; Frost; 6775; our lectotype designation label [present designation] (FMNH). Paralectotype (male): Massachusetts, Framingham; Frost; 6777; our paralectotype designation label [present designation] (FMNH) [specimen missing aedeagus].
Colpodota avia Casey, 1910: 154 syn. n.
Colpodota puritana Casey, 1910: 154 syn. n. Lectotype (male): Massachusetts *puritana* Casey; Type USNM 38994; Casey bequest 1925; our lectotype designation label [present designation] (NMNH). Paralectotypes (4): Massachusetts: *puritana* Casey; Type USNM 38994; Casey bequest 1925; our paralectotype designation label [present designation] 1 male, 3 females (NMNH).

##### Material examined.

(Type material – see above). CANADA: ON: *Hald.-Norfolk Reg*., Backus Woods, Wetland trail, 42°39'54"N, 80°29'34"W, sugar maple dom. mesic forest, sift litter, 2.iv.2010, A. Brunke, 3 (DEBU), Backus Woods, 4.x.2010, 1 (DEBU), Backus Woods, north block, 42°40'7"N, 80°29'34"W, ex. sifted litter, berlese, 23.iv.2011, Brunke and Marshall, 1 (DEBU), Cronmiller Prop., ~6km W St. Williams, 42°40'20"N, 80°29'29"W, forest, sand ridge, malaise pans, 17 to 31.v.2011, Brunke & Paiero, 1 (DEBU), Cronmiller Prop., ~6km W St. Williams, 42°40'21"N, 80°29'26"W, forest, berlese vernal pool litter, 17.v.2011, A. Brunke, 1 (DEBU), Turkey Point Prov. Pk., site 1, 42°41'48"N, 80°19'48"W, forest, sift tree hole litter, 12.x.2011, A. Brunke, 1 (DEBU); *Haliburton Co*., 10 km SE Dorset, 45.16, -78.84, vernal pool litter (previously wet), 19.vi.2011, S. Kullik, 1 (DEBU), same data except: 45.17 -78.82, 17.x.2009, 1 (DEBU); *Northumberland Co*.**,** Peter’s Woods PNR, 44°7'27"N, 78°2'21"W, ex. cold wet moss on rocks and edge of spring, 15.ix.2011, A. Brunke, 1 (DEBU); *Prescott and Russell United Co*., Alfred Bog, berlese litter, forest trail, 17.vii.1982, L. LeSage, 2 (CNC).

##### Distribution.

Canada: ON, NS; USA: IN, MA, RI (Blatchley 1910; Majka et al. 2008 (as *Atheta avia*); Majka and Klimaszewski 2010). Native.

##### Comments.

In an online catalog of North American Athetini (Gusarov 2003b), *Acrotona avia* is listed as a synonym of *Acrotona subpygmaea*. In Majka et al. (2008), *Acrotona avia* (Casey) was provisionally maintained as a valid species because one of us (JK) was unable to study the aedeagus of the only male syntype of *Acrotona subpygmaea*, which was missing or overcleared. After examination of additional material, we have discovered that the female syntype of *Acrotona subpygmaea* is very distinctive for its deeply emarginate apex of sternite VIII (Fig. 131) and shape of the spermatheca (Fig. 129), characteristics shared by the female syntypes of *Acrotona avia*. Additionally, both species do not differ externally. Therefore, to provide taxonomic stability for this common species, we here synonymize *Acrotona avia* (Casey) with *Acrotona subpygmaea* (Bernhauer) and designate a lectotype for the latter species. Majka et al. (2008) synonymyzed *Acrotona puritana* (Casey) with *Acrotona avia* (synonymy confirmed here), which now becomes a synonym of *Acrotona subpygmaea* (Bernhauer). We here designate a lectotype for *Acrotona puritana* (Casey). Specimens reported from New Brunswick and illustrated as *Acrotona subpygmaea* in Klimaszewski et al. (2005b) represent an undescribed species that will be treated in a future publication.

There are some Canadian specimens currently identified as *Acrotona subpygmaea* that possess very short elytra and slightly different sexual characters (R. Webster and J. Klimaszewski *unpublished data*) including one Ontario female [Backus Woods, Wetland trail, 42°39'54"N, 80°29'34"W, sugar maple dom. mesic forest, sift litter, 2.iv.2010]. Therefore, we recommend that identifications of *Acrotona subpygmaea* be based on the distinctive sexual characteristics of either sex (Figs 125–131) until the Nearctic diversity of this genus is more adequately known. *Acrotona subpygmaea* is a common species occurring in a variety of forest litter microhabitats and has been collected in both spring and fall. We expect this species to occur broadly across northeastern North America.

#### Alevonota gracilenta

(Erichson, 1839) New North American Record

http://species-id.net/wiki/Alevonota_gracilenta

**Figures 49–54.**
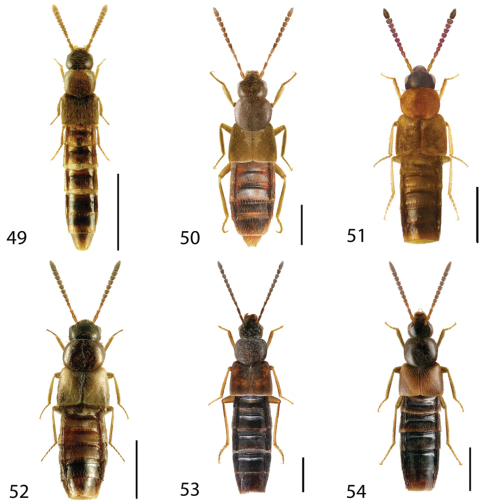
Dorsal habitus of: **49** *Alevonota gracilenta* (Erichson) **50** *Aloconota sulcifrons* (Stephens) **51** *Atheta capsularis* Klimaszewski **52** *Atheta aemula* (Erichson) **53** *Atheta borealis* Klimaszewski & Langor **54** *Atheta circulicollis* Lohse. Scale 1mm.

**Maps 49–52.**
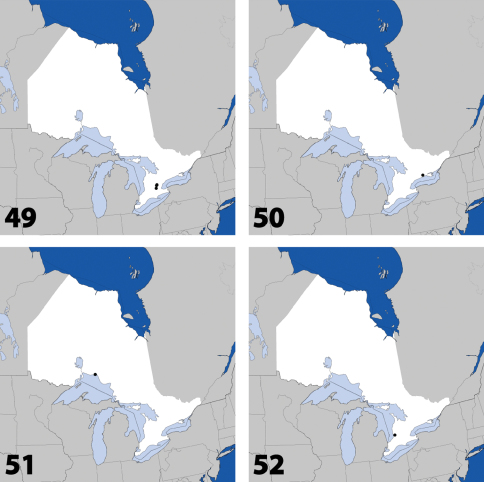
Distribution in Ontario of: **49** *Alevonota gracilenta* (Erichson) **50** *Aloconota sulcifrons* (Stephens) **51** *Atheta capsularis* Klimaszewski **52** *Atheta aemula* (Erichson).

Figs 49 132–134 Map 49 spermatheca in Assing and Wunderle (2008)

##### Material examined.

CANADA: ON:*Waterloo Reg*.,Blair, Whistle Bare Rd. and Township Rd.1, 43.372 -80.362, soybean field, pitfall trap, 29.vi.2010, A. Brunke, 2 (DEBU); *Wellington Co*.,Eramosa, hedgerow, pitfall, 4.v.2010, A. Brunke, 1 (DEBU), same data except: 13.vii.2010, 1 (DEBU), Guelph, hedgerow, pitfall, 19.v.2009, 1 (DEBU), same data except: 1.ix.2009, 1 (DEBU).

##### Distribution.

Canada: ON; widespread in western Palaearctic (Assing and Wunderle 2008). Adventive in Canada.

##### Comments.

*Alevonota gracilenta* is recorded here for the first time in North America as an adventive species. It is rather easily recognized in North America by the narrow, linear habitus, small eyes and distinctive aedeagus with a long flagellum (Fig. 132).

*Alevonota gracilenta* apparently prefers a wide range of unforested habitats in its native range but is usually only collected in small numbers and using passive traps (Assing and Wunderle 2008). It was suggested that known specimens represent dispersing individuals and that the real habitat preferences of this species remain unknown, but are possibly subterranean (Assing and Wunderle 2008). The accidental introduction of this obscure Palaearctic species to North America is surprising and may be quite recent as all known specimens are from 2009–2010 and from two contiguous counties in southern Ontario. A specimen identified as *Alevonota* by G.A. Lohse from Colorado is deposited in the CNC (A. Davies *pers. comm*.) and study of this specimen may reveal that native *Alevonota* species occur in North America.

#### Aloconota sulcifrons

(Stephens, 1832) New Ontario Record

http://species-id.net/wiki/Aloconota_sulcifrons

Fig. 50 Map 50 genitalia in Klimaszewski et al. (2011)

##### Material examined.

CANADA: ON:*Northumberland Co*., Peter’s Woods PNR, 44°7'27"N, 78°2'21"W, forest, 12.viii.2011, A. Brunke, 1 (DEBU).

##### Distribution.

Canada: ON, QC, NB, NL; USA: AL, IL, IN, KY, MO, NH, NY, TN, VA, WA, WV; widespread in Palaearctic region, possibly cosmopolitan (Fauvel 1889; Klimaszewski and Peck 1986 (*as A. insecta*); Gusarov 2003a; Smetana 2004; Majka and Klimaszewski 2008c; Webster et al. 2009; Klimaszewski et al. 2011). Adventive in Canada.

#### Atheta capsularis

Klimaszewski, 2005 New Ontario Record

http://species-id.net/wiki/Atheta_capsularis

Fig. 51 Map 51 genitalia in Klimaszewski et al. (2005b)

##### Material examined.

CANADA: ON:*Thunder Bay Distr*., Neys Provincial Park, campground area 2, 48°47'17"N, 86°37'32"W, forest, dung pans, 16 to 19.vii.2002, M. Buck, 1 (DEBU).

##### Distribution.

Canada: YT, ON, QC, NB, NL (Klimaszewski et al. 2005b; Klimaszewski et al. 2007b; Klimaszewski et al. 2011). Native.

#### Atheta (Atheta) aemula

(Erichson, 1839) New Ontario Record

http://species-id.net/wiki/Atheta_aemula

Fig. 52 Map 52 genitalia in Gusarov (2003a)

##### Material examined.

CANADA: ON:*Huron Co*., Brucefield, hedgerow, pitfall, 11.v.2009 (1), 8.vi.2009 (1), A. Brunke (DEBU).

##### Distribution.

Canada: ON, QC, NB; USA: CA, IA, KS, MA, MS, NC, NH, NJ, NY, PA, TX (Bernhauer 1907; Bernhauer 1909; Gusarov 2003a; Webster et al. 2009). Native.

#### Atheta (Atheta) borealis

Klimaszewski & Langor, 2011 New Ontario Record

http://species-id.net/wiki/Atheta_borealis

**Maps 53–56.**
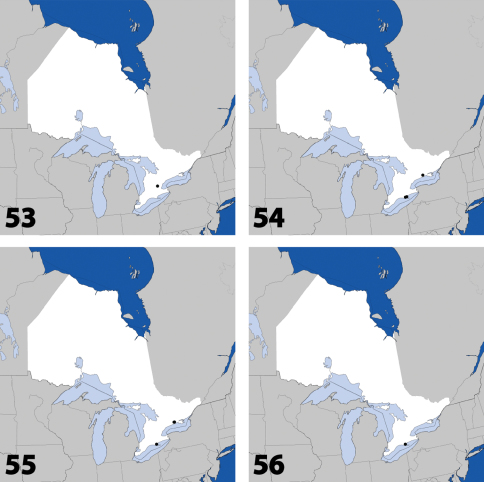
Distribution in Ontario of: **53** *Atheta borealis* Klimaszewski and Langor **54** *Atheta circulicollis* Lohse **55** *Atheta particula* (Casey) **56** *Atheta burwelli* (Lohse).

Fig. 53 Map 53 genitalia in Klimaszewski et al. (2011)

##### Material examined.

CANADA: ON:*Wellington Co*., Arkell, wet sedge meadow, sweep, 7.x.1993, C.S. Blaney, 1 (DEBU).

##### Distribution.

Canada: ON, NL (Klimaszewski et al. 2011). Native.

#### Atheta (Atheta) circulicollis

Lohse, 1990 New Ontario Record

http://species-id.net/wiki/Atheta_circulicollis

Fig. 54 Map 54 genitalia in Klimaszewski et al. (2011)

##### Material examined.

CANADA: ON:*Hald.-Norfolk Reg*., Cronmiller Prop., ~6km W St. Williams, 42°40'18"N, 80°29'24"W, forest, site 2, malaise pans, 17 to 31.v.2011, Brunke & Paiero, 1 (DEBU), Turkey Point Prov. Park, 42°41'48"N, 80°19'48"W, forest, malaise pans, 31.v to 15.vi.2011, Brunke & Paiero, 1 (DEBU); *Northumberland Co*., Peter’s Woods PNR, 44°7'27"N, 78°2'21"W, forest, malaise, 19.v to 1.vi.2011, Brunke & Paiero, 1 (DEBU), same data except: back woods, forest, malaise pans, 1 to 16.vi.2011, 1 (DEBU).

##### Distribution.

Canada: ON, QC, NB, NL (Lohse et al. 1990; Klimaszewski et al. 2011; Webster et al. 2012). Native.

##### Comments.

This species was previously known only from relatively northern, forested localities in Canada, including near the tree line in Quebec (Lohse et al. 1990). The collections made from southern Ontario forests are surprising and suggest a much broader distribution in northeastern North America.

#### Atheta (Datomicra) particula

(Casey, 1910) New Ontario Record

http://species-id.net/wiki/Atheta_particula

**Figures 55–60.**
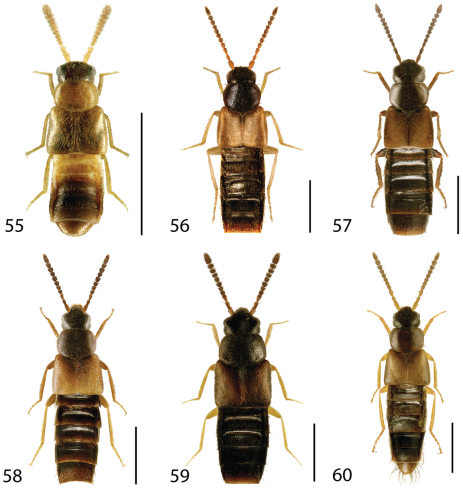
Dorsal habitus of: **55** *Atheta particula* (Casey) **56** *Atheta burwelli* (Lohse) **57** *Atheta campbelli* Lohse **58** *Atheta pseudocrenuliventris* Klimaszewski **59** *Atheta terranovae* Klimaszewski & Langor **60** *Atheta savardae* Klimaszewski & Majka. Scale 1mm.

Fig. 55 Map 55 genitalia in Klimaszewski et al. (2005)

##### Material examined.

CANADA: ON:*Hald.-Norfolk Reg*., Cronmiller Prop., ~6 km W St. Williams, 42°40'18"N, 80°29'24"W, forest, nr. vernal pools, malaise pans, 31.v to 15.vi.2011, Brunke & Paiero, 2 (DEBU), Cronmiller Prop., ~6 km W St. Williams, 42°40'18"N, 80°29'24"W, forest, 17.viii.2011, A. Brunke, 1 (DEBU); *Northumberland Co*., Barr Property, ~ 7km NE Centreton, 44°7'48"N, 77°59'3"W, old field, malaise pans, 1 to 16.vi.2011, Brunke & Paiero, 1 (DEBU), Peter’s Woods PNR, 44°7'26"N, 78°2'31"W, forest, on fungus, 12.viii.2011, S.M. Paiero, 1 (DEBU), Peter’s Woods PNR, 44°7'26"N, 78°2'31"W, forest, 12.viii.2011, A. Brunke, 1 (DEBU).

##### Distribution.

Canada: ON, QC, NB, NS; USA: NY, RI (Moore and Legner 1975; Klimaszewski et al. 2005b; Majka and Klimaszewski 2010). Native.

#### Atheta (Dimetrota) burwelli

(Lohse, 1990) New Ontario Record

http://species-id.net/wiki/Atheta_burwelli

Fig. 56 Map 56 genitalia in Klimaszewski et al. (2011)

##### Material examined.

CANADA: ON:*Hald.-Norfolk Reg*., Cronmiller Prop., ~6km W St. Williams, 42°40'20"N, 80°29'29"W, site 2, forest, malaise pans, 17 to 31.v.2011, Brunke & Paiero, 2 (DEBU).

##### Distribution.

Canada: YT, ON, QC, NB, NL (Lohse et al. 1990; Klimaszewski et al. 2008; Majka and Klimaszewski 2008a; Klimaszewski et al. 2011). Native.

#### Atheta (Dimetrota) campbelli

(Lohse, 1990) New Ontario Record

http://species-id.net/wiki/Atheta_campbelli

**Maps 57–60.**
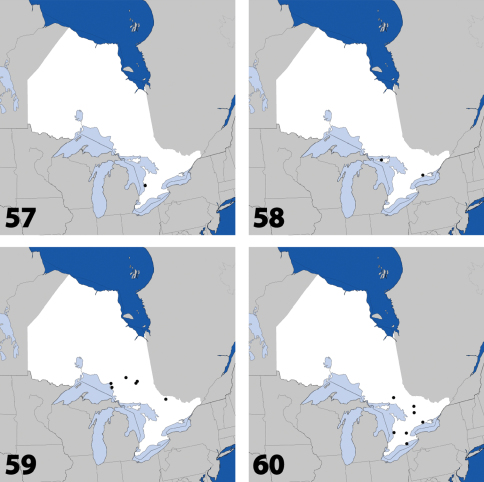
Distribution in Ontario of: **57** *Atheta campbelli* Lohse **58** *Atheta pseudocrenuliventris* Klimaszewski **59** *Atheta terranovae* Klimaszewski and Langor **60** *Atheta savardae* Klimaszewski and Majka.

Fig. 57 Map 57 genitalia in Klimaszewski et al. (2011)

##### Material examined.

CANADA: ON:*Huron Co*., Auburn, hedgerow, pitfall, 26.v.2010, A. Brunke, 1 (DEBU).

##### Distribution.

Canada: YT, ON, NL; USA: AK (Lohse et al. 1990; Klimaszewski et al. 2011). Native.

#### Atheta (Dimetrota) pseudocrenuliventris

Klimaszewski, 2005 New Ontario Record

http://species-id.net/wiki/Atheta_pseudocrenuliventris

Fig. 58 Map 58 genitalia in Klimaszewski et al. (2005b)

##### Material examined.

CANADA: ON:*Manitoulin Distr*., Manitoulin Is., Kip Fleming Tract, ~8km SW Gore Bay, 45°52'13"N, 82°32'31"W, oak savannah/alvar, RET over burrow, 15.vi to 16.vii.2010, Marshall et al., 1 (DEBU); *Northumberland Co*., Peter’s Woods PNR, 44°7'27"N, 78°2'21"W, front woods, forest, malaise pans, 16 to 27.vi.2011, Brunke & Paiero, 1 (DEBU).

##### Distribution.

Canada: YT, ON, NB, NS, NL (Klimaszewski et al. 2005b; Majka et al. 2006; Klimaszewski et al. 2008a; Klimaszewski et al. 2011). Native.

#### Atheta (Dimetrota) terranovae

Klimaszewski & Langor, 2011 New Ontario Record

http://species-id.net/wiki/Atheta_terranovae

Fig. 59 Map 59 genitalia in Klimaszewski et al. (2011)

##### Material examined.

CANADA: ON:*Algoma Distr*., Lake Superior Prov. Pk., 2.ix.1980, leg. R. Baranowski, 6 (MZLU), same data except: 3.ix.1980, 1 (MZLU), 6.ix.1980, 4 (MZLU), Michipicoten River (south of Wawa), 5.ix.1980, leg. R. Baranowski, 7 (MZLU), same data except: 8.ix.1980, leg. R. Baranowski, 2 (MZLU); *Nipissing Distr*., Algonquin Prov. Park nr. Brent, 20.viii.1980, leg. R. Baranowski, 5 (MZLU), same data except: 21.viii.1980, 1 (MZLU); *Sudbury Distr*., 30 km W of Foleyet, 30.viii.1980, leg. R. Baranowski, 2 (MZLU), Gogama, Mattagami River, 24.viii.1980, leg. R. Baranowski, 1 (MZLU), same data except: 27.viii.1980, 4 (MZLU), Mattagami, 25.viii.1980, leg. R. Baranowski, 1 (MZLU), same data except: 27.viii.1980, 4 (MZLU).

##### Distribution.

Canada: YT, ON, NB, NL (Klimaszewski et al. 2011; Klimaszewski et al. 2012; Webster et al. 2012). Native.

##### Comments.

The above Ontario collections of this recently described species suggest a transcontinental distribution in Canada.

#### Atheta (Metadimetrota) savardae

Klimaszewski and Majka, 2007 New Ontario Record

http://species-id.net/wiki/Atheta_savardae

Fig. 60 Map 60 genitalia in Klimaszewski and Majka (2007b)

##### Material examined.

CANADA: ON:*Greater Sudbury Div*., Sudbury, Laurentian Univ. Campus, 46°27'38"N, 80°57'33"W, forest, pitfall trap, 1.ix.2010 (2), 24.ix.2010 (4), 27.ix.2010 (4), 29.ix.2010 (4), 4.x.2010 (4), 6.x.2010 (1), 8.x.2010 (1), J.S. Jackson (DEBU); *Hald.-Norfolk Reg*.,Turkey Point Prov. Park, 42°41'48"N, 80°19'48"W, forest, on fungi, 12.x.2011, S.M. Paiero, 1 (DEBU); *Haliburton Co*., Dorset, 18 km S of Frost Centre, fungus, 19.ix.2008, S. Kullik, 1 (DEBU); *Huron Co*., Auburn, hedgerow, pitfall, 10.ix.2010, A. Brunke, 1 (DEBU); *Nipissing Distr*., Algonquin Prov. Park, Swan Lake station, Scott Lk., 45°29'15" 78°43'20"W, shore site, pan traps, 4.vii.1995, S. A. Marshall, 1 (DEBU); *Northumberland Co*.,Peter’s Woods PNR, 44°7'26"N, 78°2'31"W, forest, 6.x.2011, A. Brunke, 1 (DEBU); *Wellington Co*., Arkell, field vegetation, 1.x.1993, C. Krupke, 1 (DEBU).

##### Distribution.

Canada: ON, QC, NB, NS, NL (Klimaszewski and Majka 2007b; Webster et al. 2009; Klimaszewski et al. 2011). Native.

##### Comments.

This species appears to be associated with decaying fungi in forested habitats as all known specimens with microhabitat data were collected this way.

#### Atheta (Microdota) alesi

Klimaszewski & Brunke sp. n.

urn:lsid:zoobank.org:act:76CCEC54-23E7-4DB2-B34F-B4D14BFDE7BC

http://species-id.net/wiki/Atheta_alesi

**Figures 61–66.**
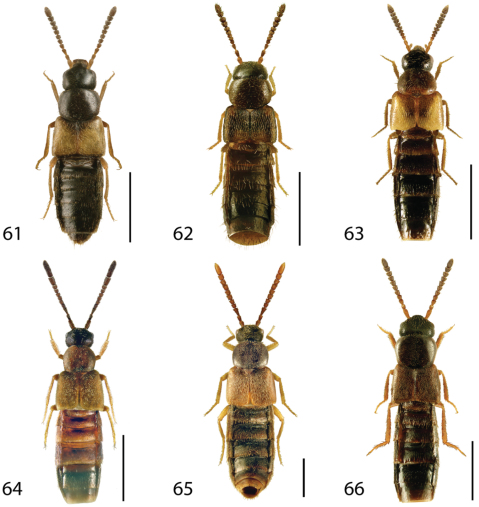
Dorsal habitus of: **61** *Atheta alesi* Klimaszewski & Brunke sp. n. **62** *Atheta festinans* (Erichson) **63** *Atheta nescia* (Casey) **64** *Callicerus obscurus* Gravenhorst **65** *Callicerus rigidicornis* (Erichson) **66** *Dinaraea backusensis* Klimaszewski & Brunke sp. n.Scale 1mm.

**Maps 61–64.**
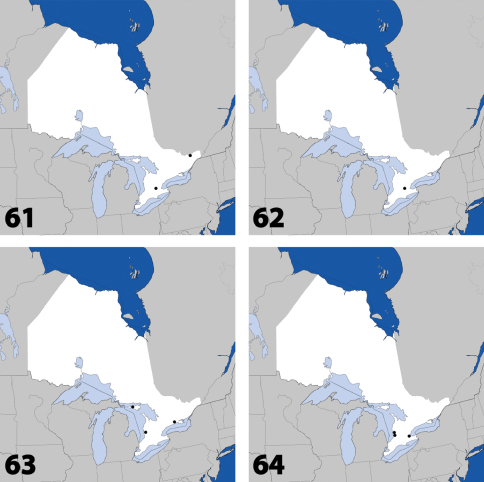
Distribution in Ontario of: **61** *Atheta alesi* Klimaszewski & Brunke sp. n. **62** *Atheta festinans* (Erichson) **63** *Atheta nescia* (Casey) **64** *Callicerus obscurus* Gravenhorst.

Figs 61 135–141 Map 61

##### Type locality.

Canada, Ontario, Ottawa Div., Ottawa, Central Experimental Farm, *Marmota* burrow.

##### Type material.

Holotype (male): CANADA: ON: Ottawa, Centr. Exp. Farm, *Marmota* burrows, 20.iv.2009, A. Smetana leg. (LFC).

Paratypes (6 males, 8 females): 13 with same data as holotype: (2 male, 5 female, CNC; 4 male, 2 female, LFC); *Waterloo Reg*., Blair, 43.37 -80.39, hedgerow, canopy trap, 19.v.2009, A. Brunke, 1 female (DEBU).

##### Diagnosis.

This species may be distinguished from all other *Atheta (Microdota)* species by the following combination of characters: body dark brown with legs, 2–3 basal antennomeres and elytra yellowish; forebody strongly glossy and with microsculpture; distal antennomeres only moderately transverse; male tergite VIII with distinctive shallow and wide emargination (Fig. 137), median lobe of aedeagus in lateral view with large bulbus and straight tubus (Fig. 136), internal sac in lateral view with distinctive, large, curved sclerite that is bifurcate basally (Fig. 136); and spermatheca S-shaped, with elongate, tubular capsule that bears a moderately long and broad apical invagination, stem sinuate and apically looped (Fig. 139).

##### Description.

Body small, length 2.4–2.6 mm, narrowly subparallel, forebody with strong meshed microsculpture and strongly glossy, abdomen strongly glossy and with moderately sparse pubescence; head, pronotum and abdomen dark brown, elytra, legs and antennomeres 2–3 yellowish; head subquadrate, flattened and slightly impressed medially, with postocular area at least as long as diameter of eye, eyes large and slightly protruding, pubescence directed inwards in central part of disc; antennae slender, antennomeres 1–3 strongly elongate, 4–5 subquadrate, 6–10 moderately transverse, apical antennomere strongly elongate, longer than 9–10 combined; pronotum moderately transverse, margined laterally and posteriorly, pubescence radiating laterad and obliquely posteriad from the midline of disc, with 4 macrosetae close to lateral margin; elytra slightly elongate, at suture longer than pronotum, pubescence directed obliquely latero-posteriad; abdomen subparallel, tergites III to V with basal impression; legs moderately elongate.

Male. Tergite VIII truncate apically and with shallow, wide emargination (Fig. 137); sternite VIII rounded apically or sometimes slightly pointed medially (Fig. 138); median lobe of aedeagus with large, broad bulbus and short triangular tubus in parameral view; in lateral view, tubus straight ventrally and narrowly rounded at apex (Fig. 136); internal sac in abparameral view with distinct structures as illustrated in Fig. 135, internal sac in lateral view with distinctive curved sclerite that is bifurcate basally (Fig. 136).

Female. Tergite VIII truncate apically (Fig. 140); sternite VIII rounded and slightly pointed medially (Fig. 141); spermatheca S-shaped, with elongate, tubular capsule that bears a moderately long and broad apical invagination, stem sinuate and apically looped (Fig. 139).

##### Distribution.

*Atheta alesi* is currently only known from Ontario but is expected to occur broadly across eastern North America.

##### Bionomics.

Nearly all specimens were collected from debris in groundhog (*Marmota monax* (L.)) burrows. *Atheta alesi* may be another member of the rich insect assemblage associated with groundhog burrows but further collections in this microhabitat are needed to confirm this. Although one specimen was collected in a raised pan trap placed in an agricultural hedgerow, other groundhog-associated staphylinids were collected in this series including *Aleochara ocularis* Klimaszewski and *Bisnius pugetensis* (Hatch).

##### Etymology.

This species is named in honor of Dr. Aleš Smetana, Ottawa, Ontario, Canada, in recognition of his excellent collections from groundhog (*Marmota monax* (L.)) burrows, which have revealed many interesting species that may be restricted to this microhabitat (e.g. species in Klimaszewski 1984, Smetana 1971, 1995).

##### Comments.

This species is tentatively assigned to the subgenus *Microdota* based on the following combination of characters present in other Canadian species: small body size, antennomeres 6–10 subquadrate to moderately transverse, Y-shaped ligula, simply formed median lobe of the aedeagus and overall shape of the spermatheca. *Atheta (Microdota) alesi* is most similar externally and in sexual characters to the type species of *Microdota*, *Atheta (Microdota) amicula* (Stephens), which has become introduced into North America. The new species can be separated from *Atheta amicula* by the longer apical antennomeres (strongly transverse in *Atheta amicula*), the straight ventral surface of the tubus in lateral view, the differently shaped sclerites of the internal sac in lateral view and the narrower capsule of the spermatheca. The sexual characters of *Atheta amicula* are illustrated in Klimaszewski et al. (2011).

#### Atheta (Microdota) festinans

(Erichson, 1839)

http://species-id.net/wiki/Atheta_festinans

Fig. 62 Map 62 genitalia in Gusarov (2003a)

##### Material examined.

CANADA: ON:*Waterloo Reg*., Blair, 43.374 -80.397, hedgerow, pitfall trap, 16.vi.2009, A. Brunke, 1 (DEBU).

##### Distribution.

Canada: ON; USA: AZ, CT, IA, IN, KS, NY, PA, RI (Bernhauer 1907; Gusarov 2003a). Native.

##### Comments.

This species was previously reported from Ontario by Bernhauer (1907) and Indiana and Michigan by Moore and Legner (1975) but these records were not among those verified by Gusarov (2003a) in his revision of this species concept. We therefore provide confirmation that this species occurs in Canada. Gusarov (2003a) remarked that all specimens seen of this species were females and suggested that this species may be parthenogenetic. Congruently, the Ontario specimen is also a female.

#### Atheta (Pseudota?) nescia

(Casey, 1910) New Ontario Record

http://species-id.net/wiki/Atheta_nescia

Fig. 63 Map 63 genitalia in Klimaszewski and Winchester (2002) (as *Atheta vancouveri*)

##### Material examined.

CANADA: ON:*Huron Co*., Auburn, hedgerow, 26.v.2010, A. Brunke, 1 (DEBU); *Manitoulin Distr*., Manitoulin I., Kip Fleming Tract, 8 km SW Gore Bay, 45°52'13"N, 82°32'31"W, oak savannah/alvar, pans nr. log, 23.viii to 30.viii.2010, Marshall et al., 2 (DEBU);same data except:malaise pans, 12.vi to 16.vi.2010, 1 (DEBU); *Northumberland Co*., Barr prop., 7 km NE Centreton, site 1, 44°7'40"N, 77°58'57"W, savannah, malaise pans, 16.vi to 27.vi.2011, Brunke and Paiero, 1 (DEBU).

##### Distribution.

Canada: BC, ON (Klimaszewski and Winchester 2002 [as *Atheta vancouveri* Klimaszewski]). Native.

##### Comments.

The specimens form Ontario agree in most characteristics with British Columbian specimens of *Atheta nescia* except for the less robust antennae, particularly in males, and the median lobe in lateral view, with a slightly narrower tubus and more rounded apex (for illustrations of the genitalia of *Atheta nescia* see Figs 51–53 in Klimaszewski and Winchester 2002 under the synonymic name *Atheta vancouveri* Klimaszewski). The spermathecae of the two species are similarly shaped. Therefore we tentatively associate the Ontario specimens with *Atheta nescia* but more specimens are needed from a broader distributional range to fully establish their identity.

The Ontario specimens were captured in sparsely treed, open habitats including savannahs and an agricultural hedgerow. Similarly, the specimens of *Atheta nescia* collected in British Columbia were primarily collected in clear-cut forests (Klimaszewski and Winchester 2002, as *Atheta vancouveri*).

#### Callicerus obscurus

Gravenhorst, 1802 New Canadian Record

http://species-id.net/wiki/Callicerus_obscurus

Figs 64 142–143 Map 64

##### Material examined.

CANADA: ON:*Hamilton Div*., Hamilton, 15.v.1985, M. Sanborne, 1 (CNC); *Huron Co*., Brucefield, hedgerow, pitfall, 11.v.2009, A. Brunke, 1 (DEBU), same data except: 22.vi.2009, 1 (DEBU), Auburn, hedgerow, pitfall, 11.v.2010, A. Brunke, 1 (DEBU).

##### Distribution.

Canada: ON; western Palaearctic (Assing 2001; Gusarov 2003b). Adventive in Canada.

##### Comments.

*Callicerus obscurus* is recorded from Canada for the first time based on Ontario specimens mostly collected in agricultural hedgerows. Gusarov (2003b) first reported this species from North America in an online catalog of North American Athetini based on specimens collected in Ontario (V. Gusarov, *pers. comm*). The ‘undescribed *Callicerus* s.str.’ from Ontario groundhog burrows mentioned by Ashe (in Newton et al. 2000) may in fact be this adventive species. Therefore, all *Callicerus* in North America may be introduced. Males of *Callicerus obscurus* are easily recognized by their extremely elongate antennomere 10. In North America, *Callicerus obscurus* may be separated externally from *Callicerus rigidicornis* by the more elongate pronotum (Fig. 64).

*Callicerus obscurus* inhabits open and forested habitats in its native range and was suggested to be largely subterranean by Assing (2001) based on highly seasonal (mostly spring) surface activity and the low numbers of individuals captured in each collection event.

#### Callicerus rigidicornis

(Erichson, 1839) New North American Record

http://species-id.net/wiki/Callicerus_rigidicornis

**Maps 65–68.**
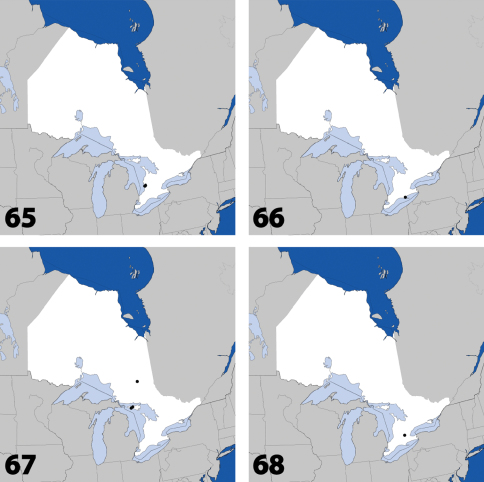
Distribution in Ontario of: **65** *Callicerus rigidicornis* (Erichson) **66** *Dinaraea backusensis* Klimaszewski & Brunke sp. n. **67** *Mocyta breviuscula* (Mäklin) **68** *Philhygra jarmilae* Klimaszewski & Langor.

Figs 65 144–145 Map 65

##### Material examined.

CANADA: ON: *Huron Co*., Auburn, hedgerow, pitfall, 11.v.2010, A. Brunke, 3 (DEBU), Benmiller, hedgerow, pitfall, 22.vi.2009, A. Brunke, 1 (DEBU).

##### Distribution.

Canada: ON; western Palaearctic (Assing 2001). Adventive in Canada.

##### Comments.

*Callicerus rigidicornis* is recorded from North America as an adventive species for the first time based on Ontario specimens collected in agricultural hedgerows. Males of this species do not have their antennomere 10 conspicuously elongate as in *Callicerus obscurus*. *Callicerus rigidicornis* is separated from *Callicerus obscurus* by the more transverse pronotum (Fig. 65). In both its native range and in Canada, this species is collected from the same habitats as *Callicerus obscurus* though the true microhabitat may be subterranean (Assing 2001).

#### Dinaraea backusensis

Klimaszewski & Brunke, sp. n.

urn:lsid:zoobank.org:act:81B9BEBD-F6ED-4893-A5C1-2094599C88CC

http://species-id.net/wiki/Dinaraea_backusensis

Figs 66 146–149 Map 66

##### Type locality.

Canada, Ontario, Haldimand-Norfolk Reg., 6 km W of Saint Williams, Backus Woods, Wetland trail, sugar maple-dominated mesic forest, 42°39'54"N, 80°29'34"W.

##### Type material.

Holotype (male): CANADA, ON:*Hald.-Norfolk Reg.*, Backus Woods, Wetland trail, 42°39'54"N, 80°29'34"W, sugar maple-dominated mesic forest, sifted litter, 2.iv.2010, A. Brunke, debu00331025 (DEBU).

##### Diagnosis.

This species may be distinguished from all other Nearctic *Dinaraea* by the following combination of characters: postocular area slightly longer than eye; pronotum trapezoidal in form and slightly (not distinctly) transverse; antennomeres 1–3 elongate, 4–7 subquadrate, 8–10 slightly transverse; elytra flat, transverse, at suture about as long as pronotum; male tergite eight with median and lateral teeth (Fig. 147); and median lobe of aedeagus of distinctive shape in lateral view (Figs 146).

##### Description.

Body narrowly subparallel, flattened, length 3.1 mm, dark brown, with legs, maxillary palpi, and basal 1–3 antennomeres yellowish brown, forebody moderately glossy with strong meshed microsculpture, abdomen strongly glossy with weaker microsculpture, pubescence moderately dense, denser on pronotum and elytra than on abdomen (Fig. 66); head transverse, impressed medially, rounded laterally, postocular area slightly longer than eye, pubescence sparse and directed mediad; antennae with antennomeres 1–3 elongate, 4–7 subquadrate, 8–10 slightly transverse; maxillary palpi with penultimate article broad and last article acicular; pronotum slightly transverse, trapezoidal, basal margin arcuate, with obtuse hind angles, broadest in apical third, flattened medially, margined, pubescence sparser than that on elytra and directed laterad on disc and forming arcuate lines, pubescence at midline directed anteriad in apical portion and posteriad in basal portion, pronotum with 4 lateral macrosetae on each side; elytra flat, transverse, subequal in length to pronotum at midline, pubescence directed straight or obliquely posteriad, punctation granulose; abdomen with tergites II-IV strongly impressed and sparsely pubescent.

Male. Tergite VIII truncate apically with two lateral teeth and two median protuberances (Fig. 147); sternite VIII with apex arcuate but slightly pointed medially (Fig. 148); median lobe of aedeagus in lateral view with moderately large bulbus and short tubus with angulate apex, ventral side of tubus weakly arcuate; internal sac in lateral view with a narrow, elongate and recurved sclerite (Fig. 146).

Female. Unknown.

##### Distribution.

At present, *Dinaraea backusensis* is known only from southern Ontario but should occur across eastern North America, at least as far north as southern Canada.

##### Bionomics.

The holotype was collected in a sugar maple dominated forest with a rich diversity of other deciduous trees by sifting deep pockets of leaf litter beside large, old logs. Other native species of *Dinaraea* have been associated with subcortical habitats (Lohse et al. 1990).

##### Etymology.

This species is named after Backus Woods, a 704-acre, old growth, Carolinian forest in Ontario, Canada where the holotypewas collected. We would like to recognize the conservation efforts of the Nature Conservancy of Canada in this region and their recent work in acquiring this property for permanent protection.

##### Comments.

Using previous literature, *Dinaraea backusensis* can be distinguished from all known Nearctic species of the genus except *Dinaraea borealis* Lohse and *Dinaraea planaris* (Mäklin) by the distinctive shape of the median lobe in lateral view (see figures in Klimaszewski et al. 2011). The male of *Dinaraea borealis* has recently been discovered (to be described in a future publication) and clearly differs in the shape of the median lobe in lateral view. The aedeagus of the lectotype of *Dinaraea planaris* is mounted in abparameral view (illustrated in Lohse and Smetana 1985) but *Dinaraea backusensis* differs from *Dinaraea planaris* by the more elongate pronotum and male tergite VIII with median and lateral teeth (unmodified and truncate apically in *Dinaraea planaris*). *Dinaraea backusensis* is most similar to the European species *Dinaraea linearis* (Gravenhorst) but differs in the following characters: median lobe in lateral view angular at apex, shorter and much broader; internal sac in lateral view with long, recurved sclerite, about as long as bulbus (much shorter and talon-like in *Dinaraea linearis*); and male tergite 8 with lateral projections longer and differently shaped than medial projections (lateral and medial projections similar in shape in *Dinaraea linearis*). Dissected specimens from Denmark (no specific locality) were examined (ZMUC). The two taxa are nearly identical externally.

#### Mocyta breviuscula

(Mäklin, 1852) New Ontario Record

http://species-id.net/wiki/Mocyta_breviuscula

**Figures 67–72.**
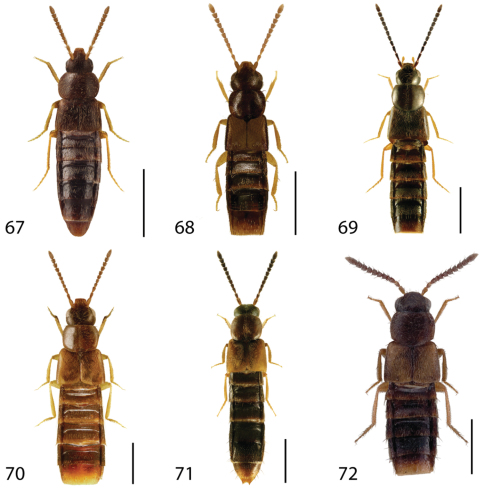
Dorsal habitus of: **67** *Mocyta breviuscula* (Mäklin) **68** *Philhygra jarmilae* Klimaszewski & Langor **69** *Philhygra laevicollis* (Mäklin) **70.** *Philhygra luridipennis* (Mannerheim) **71** *Philhygra proterminalis* (Bernhauer) **72** *Stethusa klimschi* (Bernhauer). Scale 1mm.

Fig. 67 Map 67 genitalia in Klimaszewski et al. (2011)

##### Material examined.

CANADA: ON:*Manitoulin Distr*., Manitoulin Island, Kip Fleming Tract, ~8km SW Gore Bay, 45°52'13"N, 82°32'31"W, oak savannah/alvar, sifted litter, 29.ix.2010, S.M. Paiero, 1 (DEBU), Misery Bay Prov. Nat. Res., 45°47'28"N, 82°44'58"W, alvar, 29.ix.2010, S.M. Paiero, 1 (DEBU); *Sudbury Co*., Mattagami, 24.viii.1980, leg. R. Baranowski, 1 (MZLU).

##### Distribution.

Canada: YT, BC, AB, ON, QC, NB, NS, NL; USA: AK, CA, NV (Lohse and Smetana 1985; Gusarov 2003a; Klimaszewski et al. 2007b; Klimaszewski et al. 2008a; Majka and Klimaszewski 2008c; Webster et al. 2009; Klimaszewski et al. 2011). Native.

#### Philhygra jarmilae

Klimaszewski & Langor, 2011 New Ontario Record

http://species-id.net/wiki/Philhygra_jarmilae

Fig. 68 Map 68 genitalia in Klimaszewski et al. (2011)

##### Material examined.

CANADA: ON:*Waterloo Reg*., Blair, hedgerow, pitfall, 5.x.2010, A. Brunke, 1 (DEBU).

##### Distribution.

Canada: YT, ON, NB, NL (Klimaszewski et al. 2011; Klimaszewski et al. 2012; Webster et al. 2012). Native.

##### Comments.

The specimen from southern Ontario suggests a broad, transcontinental distribution in Canada for this recently described species. It likely occurs broadly in eastern United States as well.

#### Philhygra laevicollis

(Mäklin, 1852) New Ontario Record

http://species-id.net/wiki/Philhygra_laevicollis

**Maps 69–72.**
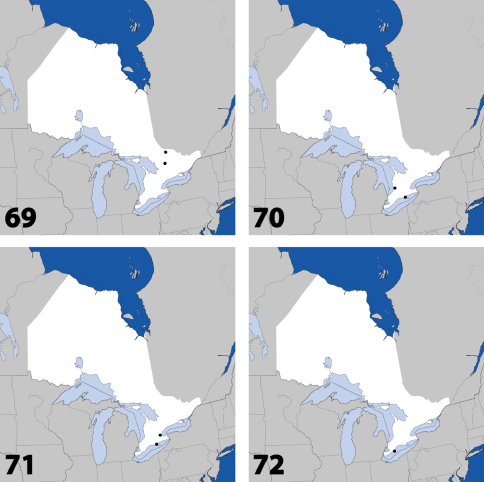
Distribution in Ontario of: **69** *Philhygra laevicollis* (Mäklin) **70** *Philhygra luridipennis* (Mannerheim) **71.** *Philhygra proterminalis* (Bernhauer) **72** *Stethusa klimschi* (Bernhauer).

Fig. 69 Map 69 genitalia in Klimaszewski et al. (2005b)

##### Material examined.

CANADA: ON:*Haliburton Co*., 9 km SE of Dorset, vernal pool litter (previously wet), 45.17–78.84, 17.vii.2009, S. Kullik, 1 (DEBU), same data except: 45.18–78.83, 17.viii.2009, 1 (DEBU); *Nipissing Distr*., Algonquin Prov. Park, nr. Brent, 19.viii.1980, R. Baranowski, 1 (MZLU), same data except: 21.viii.1980, 1 (MZLU).

##### Distribution.

Canada: BC, ON, NB, NS; USA: AK, WA (Moore and Legner 1975; Klimaszewski and Winchester 2002; Klimaszewski et al. 2005b; Majka and Klimaszewski 2008c). Native.

#### Philhygra luridipennis

(Mannerheim, 1831) New Ontario Record

http://species-id.net/wiki/Philhygra_luridipennis

Fig. 70 Map 70 genitalia in Klimaszewski et al. (2011)

##### Material examined.

CANADA: ON:*Hald.-Norfolk Reg*., Cronmiller prop., ~6km W St. Williams, 42°40'21"N, 80°29'26"W, forest, at lights, 20.vii.2011, Brunke & Paiero, 1 (DEBU); *Huron Co*., Brucefield, hedgerow, pitfall trap, 28.ix.2009, A. Brunke, 1 (DEBU).

##### Distribution.

Canada: ON, NB, NL; Palaearctic: Europe and North Africa (Smetana 2004; Klimaszewski et al. 2011; Webster et al. 2012). Holarctic or adventive species.

#### Philhygra proterminalis

(Bernhauer, 1907) New Canadian Record

http://species-id.net/wiki/Philhygra_proterminalis

Figs 71 149 Map 71

##### Material examined.

CANADA: ON:*Hald.-Norfolk Reg*., Cronmiller Prop., ~6km W St. Williams, 42°40'21"N, 80°29'26"W, forest, 31.v.2011, A. Brunke, 1 (DEBU), same data except: Lindgren funnel, 20.vii to 5.viii.2011, Brunke & Paiero, 1 (DEBU); *Hamilton Div*., Waterdown, madicolous spring, 5.viii.1985, B. Sinclair, 1 (DEBU).

##### Distribution.

Canada: ON; USA: CO, PA. Native.

#### Stethusa klimschi

(Bernhauer, 1909) New Canadian Record

http://species-id.net/wiki/Stethusa_klimschi

Fig. 72 Map 72 genitalia in Gusarov (2003c)

##### Material examined.

CANADA: ON:*Chatham-Kent Co*.,Rondeau Prov. Pk., south point trail, nr. east parking lot, 42°15'42"N, 81°50'49"W, savannah, malaise, 14.viii to 7.ix.2003, Buck and Marshall, 1 (DEBU).

##### Distribution.

Canada: ON; USA: IN, LA, MS (Gusarov 2003c). Native.

##### Comments.

This species is newly recorded from Canada, extending its known distribution considerably northward. *Stethusa klimschi* appears to be less common in Ontario than *Stethusa spuriella* (see below) as only one female specimen was found.

#### Stethusa spuriella

(Casey, 1910) New Canadian Record

http://species-id.net/wiki/Stethusa_spuriella

**Figures 73–78.**
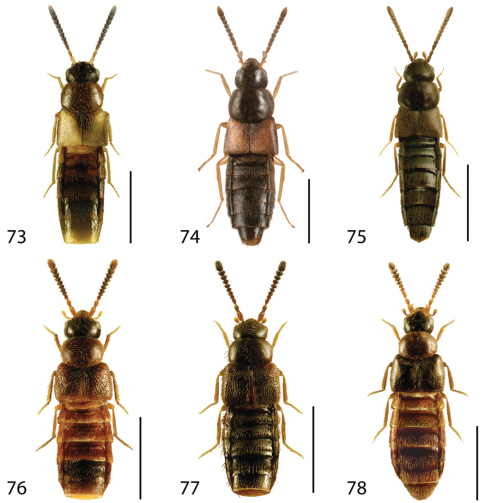
Dorsal habitus of: **73** *Stethusa spuriella* (Casey) **74** *Strigota ambigua* (Erichson) **75** *Strigota obscurata* Klimaszewski & Brunke sp. n. **76** *Trichiusa compacta* Casey **77** *Trichiusa hirsuta* Casey **78** *Trichiusa robustula* (Casey). Scale 1mm.

**Maps 73–76.**
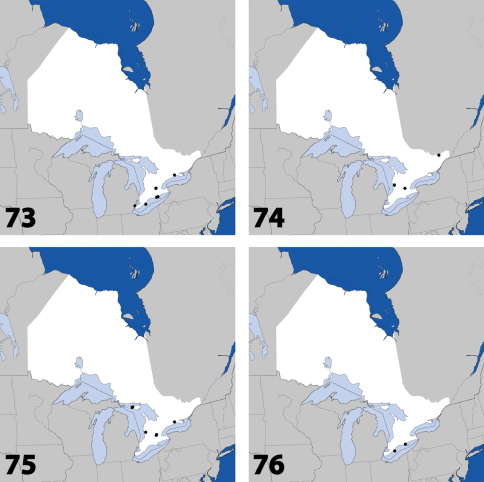
Distribution in Ontario of: **73** *Stethusa spuriella* (Casey) **74** *Strigota ambigua* (Erichson) **75** *Strigota obscurata* Klimaszewski & Brunke sp. n. **76** *Trichiusa compacta* Casey.

Fig. 73 Map 73 genitalia in Gusarov (2003c)

##### Material examined.

CANADA: ON:*Chatham-Kent Co*., Rondeau Prov. Park, south point east pkng lot, 42 15 42N, 81 50 49W, oak savannah, white pans, 29.v.2003, Buck & Paiero, 1 (DEBU); *Essex Co*., Windsor, Ojibway Prairie, burnt prairie, yellow pans, 15 to 18.v.2001, S. M. Paiero, 1 (DEBU); *Hald.-Norfolk Reg*., Cronmiller Prop., ~6km W St. Williams, 42°40'18"N, 80°29'24"W, low forest, malaise pans, 20.vii to 5.viii.2011, Brunke & Paiero, 1 (DEBU), Cronmiller Prop., ~6km W St. Williams, 42°40'20"N, 80°29'29"W, ridge forest, malaise pans, 5.vii to 20.vii.2011, Brunke & Paiero, 1 (DEBU), Turkey Point Prov. Park, site 1, 42°41'48"N, 80°19'48"W, forest, malaise pans, 5.viii to 17.viii.2011, Brunke & Paiero, 1 (DEBU), same data except: on fungus, 12.x.2011, A. Brunke, 1 (DEBU), Turkey Point Prov. Park, site 2, 42°42'28"N, 80°20'29"W, oak savannah, Berlese leaf, log and grass litter, 12.x.2011, A. Brunke, 1 (DEBU); *Northumberland Co*., Barr Property, ~ 7km NE Centreton, 44°7'44"N, 77°59'0"W, savannah, malaise pans, 26.vii to 12.viii.2011, Brunke & Paiero, 1 (DEBU); *Waterloo Reg*.,Blair, soybean field, pitfall, 23.vi.2009, A. Brunke, 1 (DEBU), Cambridge, soybean field, pitfall, 23.vi.2009, A. Brunke, 1 (DEBU).

##### Distribution.

Canada: ON; USA: DE, FL, GA, IN, MO, NJ, NY, OH, PA, (Gusarov 2003c). Native.

##### Comments.

*Stethusa spuriella* appears to be a common species in both forested and open habitats in Ontario. No Canadian specimens of the third eastern species, *Stethusa dichroa* (Gravenhorst), were discovered in our material despite its widespread occurrence in the eastern United States; it is expected to occur in southern Ontario.

#### Strigota ambigua

(Erichson, 1839) New Ontario Record

http://species-id.net/wiki/Strigota_ambigua

Fig. 74 Map 74 genitalia in Gusarov (2003a)

##### Material examined.

CANADA: ON:*Huron Co*., Auburn, soybean field, pitfall, 23.vi.2010, A. Brunke, 2 (DEBU); *Ottawa Div*., Ottawa, Centr. Exp. Farm, *Marmota* burrows, 20.iv.2009, A. Smetana, 5 (CNC); *Waterloo Reg*.,Blair, soybean field, pitfall, 23.vi.2009, A. Brunke, 1 (DEBU).

##### Distribution.

Canada: YT, ON, NS, PE, NL; USA: CA, CO, CT, IA, KS, MA, MO, NC, NJ, NM, NY, NV, TX (Bernhauer 1907; Gusarov 2003a; Majka et al. 2008; Klimaszewski et al. 2011; Klimaszewski et al. 2012). Native.

##### Comments.

This widespread species apparently prefers open habitats with well-drained soil including dunes, beaches, limestone barrens, soybean fields, old fields, open gaps in spruce forest, riverbanks and on pavement (see references under ‘distribution’). The specimens from groundhog burrows were probably overwintering there.

#### Strigota obscurata

Klimaszewski & Brunke, sp. n.

urn:lsid:zoobank.org:act:9AD7A325-4D27-411A-9748-93BF8829BD63

http://species-id.net/wiki/Strigota_obscurata

Figs 75 150–154 Map 75

##### Type locality.

Canada, Ontario, *Wellington Co*., Eramosa, Wellington Rd. 124 and 29, hedgerow nr. soybean field, 43.61 -80.21.

##### Type material.

Holotype (male): CANADA, ON: *Wellington Co*., Eramosa, Wellington Rd. 124 and 29, hedgerow, pitfall, 15.vi.2010, A. Brunke (DEBU).

Paratypes(2 males, 5 females, 7 sex unknown):labeled as the holotype, 6 sex? (DEBU); *Huron Co*., Auburn, soybean field, pitfall, 23.vi.2010, A. Brunke, 1 female, 1 male (DEBU); *Manitoulin Distr*.,Manitoulin Is., Misery Bay Prov. Nat. Res., 45°47'28"N, 82°44'58"W, alvar, malaise trap, 15.vi to 2.vii.2010, Pivar et al., debu00325236, 1 female (DEBU), Manitoulin Is., Kip Fleming Tract, 8km SW Gore Bay, 45°52'13"N, 82°32'31"W, oak savannah/alvar, under stones, 27–29.v.2010, A. Brunke, debu00323337, 1 female (DEBU); *Northumberland Co*., Barr property, 7 km NE Centreton, site 2, 44°7'48"N, 77°59'3"W, field, malaise pans, 16–27.vi.2011, Brunke & Paiero, debu01147152, 1 female (LFC), same data except: malaise, 26.vii to 12.viii.2011, debu01149211, 1 female (DEBU); *Wellington Co*., Guelph, hedgerow, 5.v.2009, A. Brunke, 1 male (LFC).

##### Diagnosis.

*Strigota obscurata* is readily separated from the other *Strigota* species by the combination of: median lobe constricted basally in parameral view (Fig. 150), male and female tergite VIII with apical margin sharply produced (Fig. 152), the dark coloration, including the legs, the body size (2.2–2.5mm) and elytra at suture distinctly shorter than the pronotum at midline (Fig. 75).

##### Description.

Body narrowly elongate, dark brown to black, with legs and/or tarsi brown, central disc of elytra sometimes with traces of reddish tinge, length 2.2–2.5 mm, moderately glossy, with dense, meshed microsculpture, pubescence short, dense and appearing somewhat silky; head convex, rounded posteriorly, postocular area at least slightly longer than the length of eye, pubescence directed towards midline of disc; antennae stout, antennomeres 1–3 strongly elongate, 4–5 subquadrate and 6–10 moderately transverse; pronotum slightly transverse, widest in basal third, pubescence directed obliquely posteriad, posteriad at midline; elytra transverse, at suture shorter than pronotum at midline, pubescence directed straight posteriad; abdomen subparallel with tergites II–IV deeply impressed basally; metatarsus with basal article as long as two following articles combined.

Male. Tergite VIII with bisinuate base and acutely produced apex, (Fig. 152); sternite VIII elongate with broad distance between base and antecostal suture, apex truncate (Fig. 153); median lobe of aedeagus in lateral view with moderately sized bulbus, tubus of median lobe slightly produced ventrad, internal sac in lateral view with several short, inconspicuous sclerites (Fig. 151); median lobe of aedeagus in ventral (parameral) view with tubus constricted basally (Fig. 150).

Female. Tergite and sternite VIII similar to those of male; spermatheca with club-shaped capsule bearing a small invagination, stem sinuate and coiled apically (Fig. 154). The spermatheca of this species is nearly identical to that of *Strigota ambigua* except for the capsule, which is more sharply deflexed and of a different shape (Fig. 154, compare with illustrations in Gusarov (2003a)).

##### Distribution.

Presently, *Strigota obscurata* is known only from Ontario but it is expected to occur widely in northeastern North America.

##### Bionomics.

*Strigota obscurata* occurs in many of the same habitats as *Strigota ambigua* and was the most commonly collected rove beetle in southern Ontario soybean fields, frequently co-occurring with the latter species (Brunke et al. *in prep*.).

##### Etymology.

The specific name is the Latin word for ‘darkened’. This is in reference to the distinct, overall darker body colorationcompared to *Strigota ambigua*, the only other eastern species of the genus.

##### Comments.

Prior to this publication there were five valid species of *Strigota* in North America: *Strigota ambigua* (Erichson) with numerous synonyms (see Gusarov (2003a)), *Strigota perplexa* Casey from Colorado, *Strigota seducens* Casey from California, *Strigota impiger* Casey from Washington and *Strigota intrudens* Casy from California. In an online catalog of Athetini, Gusarov (2003b) regarded *Strigota impiger* Casey and *Strigota intrudens* Casey as unpublished synonyms of *Strigota seducens* Casey. We have examined the types of *Strigota perplexa* Casey and *Strigota seducens* Casey. The single specimen of *Strigota perplexa* in Casey’s collection is a dissected male but features of the aedeagus could not be examined due to overclearing. The distinctive tergite 8 of *Strigota obscurata* will easily differentiate it from *Strigota perplexa* until more specimens can be examined from the type locality (Colorado, *Boulder Co.*) so that the aedeagi can be compared.

The type series of *Strigota seducens* contains 6 specimens with the following data: Cal; ‘seducens-6’, Paratype USNM 39047; Casey bequest 1925; Gusarov lect. des. 2003 [unpublished designation]; our lectotype label, present designation, [1 male, dissected, with genitalia scarcely visible] (NMNH). Same data except: ‘seducens-2’; Gusarov paralect. des. 2003 [unpublished designation]; our paralectotype label, present designation, [1 female, dissected, spermatheca not located] (NMNH). Same data as first paralectotype except: ‘seducens-3’; [1 male, dissected, aedeagus not located] (NMNH). Same except: ‘seducens-4’; [1 female, dissected, with spermatheca] (NMNH). Same except: ‘seducens-5’; [1 sex?, not dissected]. Same except: ‘seducens-Type 39047’; seducens; [1 sex?, damaged, abdomen missing].

For the purpose of nomenclatural stability, we here designate the first mentioned specimen as a lectotype and the other 5 as paralectotypes. The spermatheca of one of the paralectotypes was compared to our specimens of *Strigota obscurata* and no important differences could be found; the only available aedeagus of *Strigota seducens* was barely visible in the permanent mount and could not be compared in detail. However, *Strigota obscurata* may be differentiated from *Strigota seducens* by the combination of characters in the diagnosis and the uniformly colored elytra (light brown in centre of the disc in *Strigota seducens*). The only other eastern species of the genus, *Strigota ambigua*, is easily separated from *Strigota obscurata* by the larger size (2.4–3.0mm), less produced tergite 8 in both sexes, differently shaped aedeagus and spermatheca (Figs 150–151, 154 vs. illustrations in Gusarov (2003a)) and distinctly paler coloration of the appendages.

#### Trichiusa compacta

Casey, 1894 New Canadian Record

http://species-id.net/wiki/Trichiusa_compacta

Figs 76 155–157 Map 76

Trichiusa compacta Casey, 1894: 341. Lectotype(female): USA, DC; Type USNM 39416; Casey bequest 1925; Lectotypus, male, *Trichiusa compacta* Casey, V.I. Gusarov des. 2010 [unpublished designation]; our lectotype designation label, present designation (NMNH).

##### Material examined.

CANADA: ON: *Chatham-Kent Co*.,Rondeau Prov. Pk., 1 to 9.viii.1985, Int. trap at edge of oak forest, L. LeSage & A. Woodliffe, 1 (CNC), Rondeau Prov. Pk., South Point Trail, slough forest, leaf litter, 27.ix.2009, Brunke & Cheung, 1 (DEBU); *Hald.-Norfolk Reg*., Cronmiller prop., 6 km W St. Williams, 42°40'21"N, 80°29'26"W, forest, Berlese vernal pool litter, 17.v.2011, A. Brunke, 1 (DEBU), Cronmiller prop., 6 km W St. Williams, 42°40'20"N, 80°29'29"W, forest, sand ridge, malaise pans, 17 to 31.v.2011, Brunke & Paiero, 1 (DEBU), Cronmiller prop., 6 km W St. Williams, 42°40'21"N, 80°29'26"W, low forest, malaise, 17 to 31.v.2011, Brunke & Paiero, 1 (DEBU).

##### Distribution.

Canada: ON; USA: DC, OH. Native.

##### Comments.

The species of *Trichiusa* are currently under revision by V. Gusarov and so *Trichiusa compacta* is currently best recognized by the combination of habitus and the following sexual characters: median lobe of aedeagus in lateral view with tubus narrow, evenly subparallel and narrowly rounded apically (not sharp) (Fig. 156); spermatheca with moderately large, spherical and basally narrowed capsule bearing a deep apical invagination, stem C-shaped, looped and twisted posteriad (Fig. 157).

*Trichiusa compacta* appears to be forest inhabiting and was collected from a variety of passive traps and by sifting litter near vernal and semi-permanent forest pools.

#### Trichiusa hirsuta

Casey, 1906 New Canadian Record

http://species-id.net/wiki/Trichiusa_hirsuta

**Maps 77–79.**
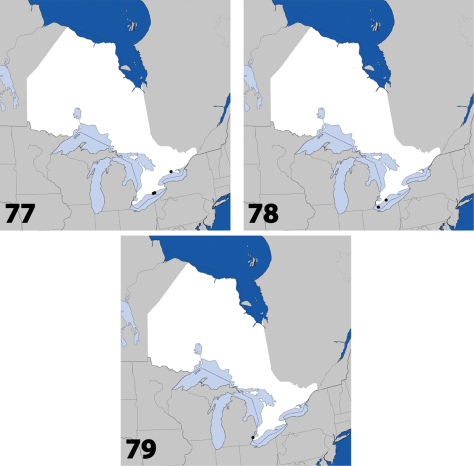
Distribution in Ontario of: **77** *Trichiusa hirsuta* Casey **78** *Trichiusa robustula* (Casey) **79** *Zyras planifer* (Casey).

Figs 77 158–160 Map 77

Trichiusa hirsuta Casey, 1906: 329. **Lectotype** (male): USA, Virginia; *hirsuta* Casey; Type USNM 39423; Casey bequest 1925; Lectotypus, male, *Trichiusa hirsuta* Casey, V.I. Gusarov des. 2011 [unpublished designation]; our lectotype designation label, present designation (NMNH).

##### Material examined.

CANADA: ON:*Hald.-Norfolk Reg*., Cronmiller prop., 6 km W St Williams, 42°40'20"N, 80°29'29"W, forest, sand ridge, malaise pan, 17.v to 31.v.2011, Brunke & Paiero, 1 (DEBU), Turkey Point Prov. Pk., site 1, 42°41'48"N, 80°19'48"W, forest, malaise pans, 17 to 31.v.2011, Brunke & Paiero, 1 (DEBU); *Northumberland Co*., Barr prop., 7 km NE Centreton, site 1, 44°7'40"N, 77°58'57W, savannah, malaise pans, 16 to 27.vi.2011, Brunke & Paiero, 1 (DEBU).

##### Distribution.

Canada: ON; USA: VA. Native.

##### Comments.

This species is currently recognizable only by the combination of habitus (Fig. 77) and the following sexual characters: median lobe of aedeagus in lateral view with tubus narrowed toward apex and sharply pointed apically (not rounded) (Figs 158–159); spermatheca with large, spherical and basally narrowed capsule bearing a small apical invagination, stem relatively straight, looped and twisted posteriad (Fig. 160).

Unlike *Trichiusa robustula*, *Trichiusa hirsuta* was collected from upland forested or semi-forested habitats on sandy soil. More collections will help elucidate the habitat requirements of this species.

#### Trichiusa robustula

Casey, 1894 New Canadian Record

http://species-id.net/wiki/Trichiusa_robustula

Figs 78 161–164 Map 78

Trichiusa robustula Casey, 1894: 343. Lectotype (male): USA, Iowa; *robustula*-8, Paratype USNM 39431; Casey bequest 1925; Lectotypus, male, *Trichiusa robustula* Casey, V.I. Gusarov des. 2011 [unpublished designation]; our lectotype designation label, present designation (NMNH).

##### Material examined.

CANADA: ON: *Chatham-Kent Co*.,Rondeau Provincial Park, beach near entrance, 3.vi.1985, in debris on beach at high water line, A. Davies & J.M. Campbell, 2 (CNC), Rondeau Provincial Pk., South Beach, 5.vi.1985, in debris on beach at high water line, A. Davies & J.M. Campbell, 1 (CNC), Rondeau Provincial Park, Lakeshore Road, 6.vi.1985, sifted grass pile and leaves, A. Davies & J.M. Campbell, 3 (CNC);*Essex Co*., East Sister I. Prov. Nat. Res., 41°49’N 82°51’W, 30.vii.2003, shore, yellow pans, S.A. Marshall, 1 (DEBU).

##### Distribution.

Canada: ON; USA: IA. Native.

##### Comments.

This species is currently recognizable only by the combination of habitus (Fig. 78) and the following sexual characteristics: median lobe of aedeagus in lateral view with tubus relatively broad, evenly subparallel and rounded apically (not sharp at apex) (Fig. 162); spermatheca with tubular capsule bearing large and deep apical invagination, stem sinuate, looped and twisted posteriad (Figs 163–164).

This species has been collected from the shoreline of the Great Lakes or from debris nearby. Further collecting is needed to determine whether or not *Trichiusa robustula* is typical of lakeshore habitat.

### Tribe Lomechusini Fleming, 1821

#### Zyras planifer

(Casey, 1894) New Canadian Record

http://species-id.net/wiki/Zyras_planifer

**Figure 79.**
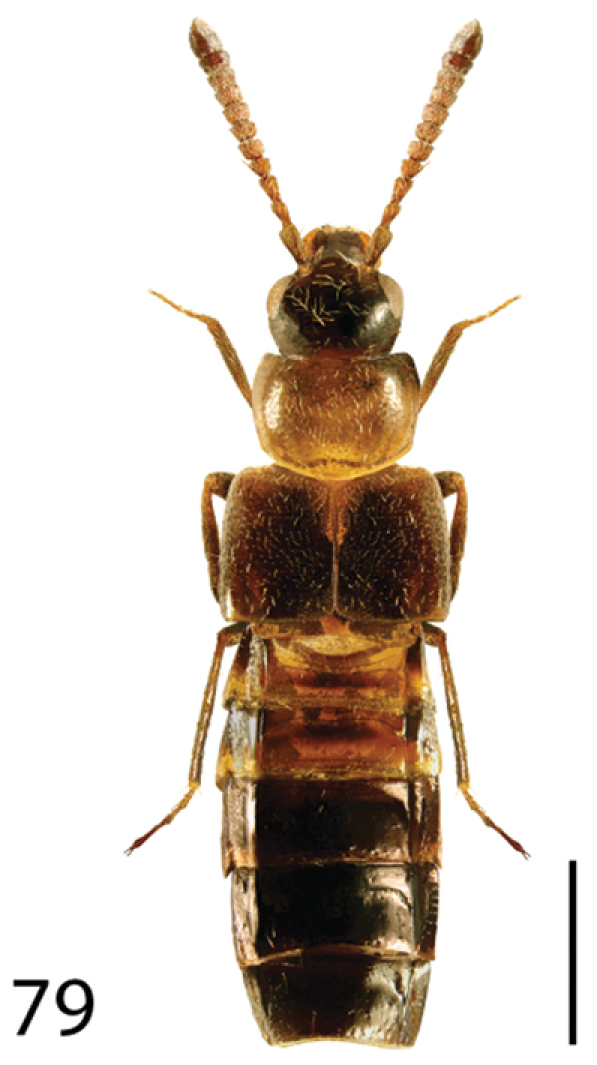
Dorsal habitus of *Zyras planifer* (Casey). Scale 1mm.

Fig. 79 Map 79 genitalia in Klimaszewski et al. (2005a)

##### Material examined.

CANADA: ON:*Essex Co*., Windsor, Ojibway Prairie, unburnt forest, yellow pans, 8 to 12.vi.2001, S.M. Paiero, 1 (DEBU).

##### Distribution.

Canada: ON; USA: DC, IN, NC (Klimaszewski et al. 2005a). Native.

**Figures 80–89.**
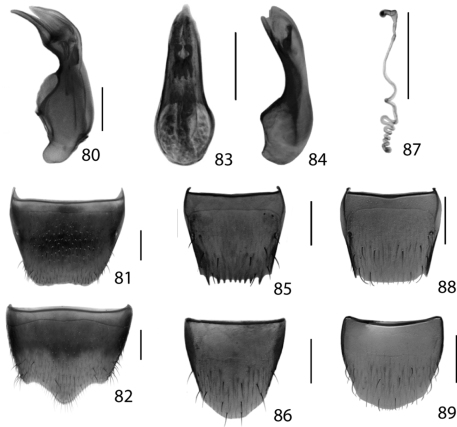
*Aleochara daviesi* Klimaszewski & Brunke sp. n.: **80** aedeagus in lateral view **81** male tergite 8 **82** male sternite 8. *Dexiogyia angustiventris* (Casey) **83** aedeagus in abparameral view **84** aedeagus in lateral view **85** male tergite 8 **86** male sternite 8 **87** spermatheca **88** female tergite 8 **89** female sternite 8. Scale 0.2 mm.

**Figures 90–102.**
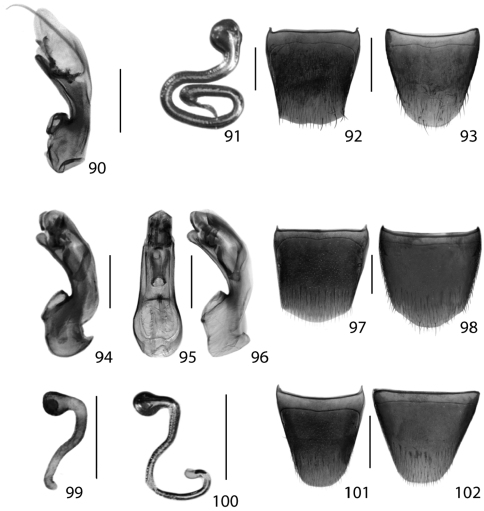
*Oxypoda rubescans* Casey **90** aedeagus lateral view. *Parocyusa americana* (Casey) **91** spermatheca **92** female tergite 8 **93** female sternite 8. *Parocyusa fuliginosa* (Casey) **94** aedeagus in lateral view [specimen from type series North Carolina] **95** aedeagus in abparameral view [Newfoundland] **96** aedeagus in lateral view [Newfoundland] **97** male tergite 8 **98** male sternite 8 **99** spermatheca [specimen from type series North Carolina] **100** spermatheca [Newfoundland] **101** female tergite 8 **102** female sternite 8. Scale 0.2 mm.

**Figures 103–115.**
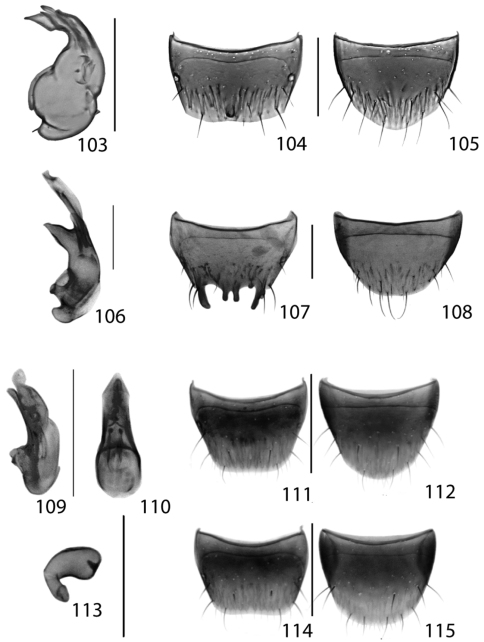
*Agaricomorpha websteri* Klimaszewski & Brunke sp. n.: **103** aedeagus lateral view **104** male tergite 8 **105** male sternite 8. *Gyrophaena caseyi* Seevers: **106** aedeagus lateral view **107** male tergite 8 **108** male sternite 8. *Thecturota pusio* (Casey) **109** aedeagus lateral view **110** aedeagus abparameral view **111** male tergite 8 **112** male sternite 8 **113** spermatheca **114** female tergite 8 **115** female sternite 8. Scale 0.2 mm.

**Figures 116–131.**
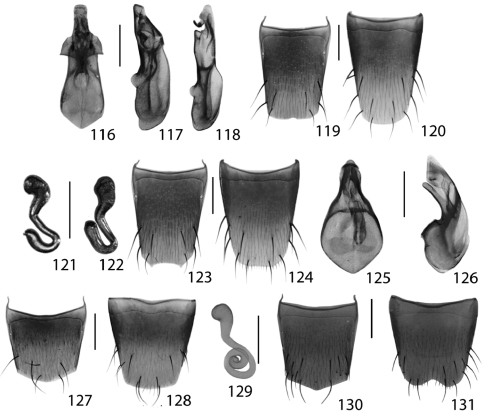
*Acrotona smithi* (Casey) **116** aedeagus abparameral view **117** aedeagus lateral view internal sac retracted **118** aedeagus lateral view internal sac everted **119** male tergite 8 **120** male sternite 8 **121–122** spermathecae **123** female tergite 8 **124** female sternite 8. *Acrotona subpygmaea* (Bernhauer): **125** aedeagus abparameral view **126** aedeagus lateral view **127** male tergite 8 **128** male sternite 8 **129** spermatheca **130** female tergite 8 **131** female sternite 8. Scale 0.2 mm.

**Figures 132–149.**
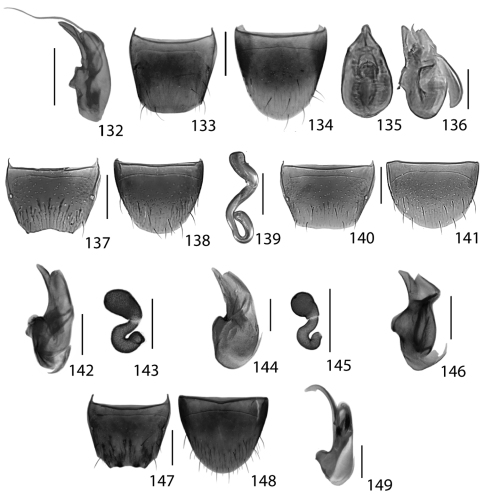
*Alevonota gracilenta* (Erichson) **132** aedeagus lateral view **133** male tergite 8 **134** male sternite 8. *Atheta (Microdota) alesi* Klimaszewski & Brunke sp. n.: **135** aedeagus abparameral view **136** aedeagus lateral view **137** male tergite 8 **138** male sternite 8 **139** spermatheca **140** female tergite 8 **141** female sternite 8. *Callicerus obscurus* Gravenhorst: **142** aedeagus lateral view **143** spermatheca. *Callicerus rigidicornis* (Erichson) **144** aedeagus lateral view **145** spermatheca. *Dinaraea backusensis* Klimaszewski and Brunke sp. n.: **146** aedeagus lateral view **147** male tergite 8 **148** male sternite 8. *Philhygra proterminalis* (Bernhauer): **149** aedeagus lateral view. Scale 0.2 mm.

**Figures 150–164.**
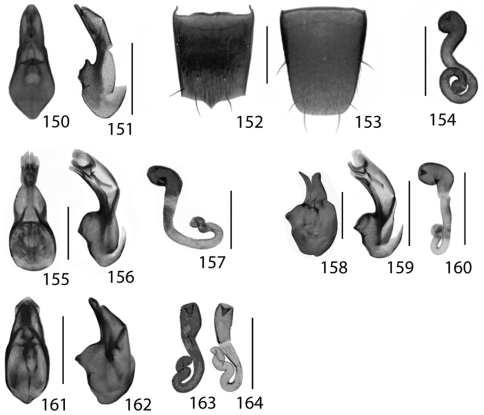
*Strigota obscurata* Klimaszewski & Brunke sp. n.: **150** aedeagus parameral view **151** aedeagus lateral view **152** male tergite 8 **153** male sternite 8 **154** spermatheca. *Trichiusa compacta* Casey **155** aedeagus abparameral view **156** aedeagus lateral view **157** spermatheca. *Trichiusa hirsuta* Casey **158** aedeagus lateral view internal sac retracted **159** aedeagus lateral view internal sac everted **160** spermatheca. *Trichiusa robustula* Casey **161** aedeagus abparameral view **162** aedeagus lateral view **163–164** spermathecae. Scale 0.2 mm.

## General discussion

This project is part of a recent effort to document the Canadian biodiversity of the large staphylinid subfamily Aleocharinae. Prior investigations have involved the faunas of Vancouver Island (BC) (Klimaszewski and Winchester 2002), Yukon Territory (Klimaszewski et al. 2008; Klimaszewski et al. 2012), ‘arctic Canada’ (Lohse et al. 1990), southeastern Quebec (Klimaszewski et al. 2007), Newfoundland (Klimaszewski et al. 2011) and the Maritime Provinces (many references *e.g*., Webster et al. 2009; Majka and Klimaszewski 2010). Identification of Ontario material was greatly facilitated by the aforementioned research but was challenging in some cases compared to that of other regions of Canada due to the presence of more ‘southern’ groups of species or genera not found elsewhere in Canada. In many cases, these specimens were left for future research involving comprehensive revisions of genera, especially those of Athetini. Once these studies are undertaken, the known diversity of Aleocharinae in Canada is expected to rise dramatically. Nevertheless, the present contribution substantially increased the known Ontario fauna by 53%.

Although the specimens studied over the course of this project were from a variety of localities and survey projects, 62% of new distributional records were derived wholly or in part form material generated from a one-year Ontario arthropod survey, a partnership between the University of Guelph Insect Collection, Nature Conservancy of Canada and Ontario Ministry of Natural Resources. We feel that the results of the present study demonstrate that species-level inventories can contribute data that significantly enrich our knowledge of Canadian biodiversity, both adventive and native. In the United States, all-taxa biological inventory projects such as the Great Smokey Mountains ATBI and the Boston Harbor Islands ATBI are making similar contributions to our knowledge of arthropod biodiversity (examples for Coleoptera: Park, Carlton et al. 2010; Davidson and Rykken 2011).

Considering the high return of discoveries made during the present study from a relatively small amount of material, it is clear that the checklist of Ontario Aleocharinae provided here represents only a preliminary but important baseline. Undoubtedly, more new species await description and several adventive species known elsewhere in eastern Canada have not yet been recorded from Ontario. We hope that this new baseline will act as a useful intermediate step towards the documentation of Canada’s arthropod biodiversity.

## Supplementary Material

XML Treatment for Deinopsis illinoisensis

XML Treatment for Aleochara (Echochara) daviesi

XML Treatment for Aleochara (Aleochara) lustrica

XML Treatment for Aleochara (Xenochara) tristis

XML Treatment for Tinotus trisectus

XML Treatment for Hoplandria klimaszewskii

XML Treatment for Hoplandria laevicollis

XML Treatment for Hoplandria laeviventris

XML Treatment for Platandria carolinae

XML Treatment for Amarochara brevios

XML Treatment for Amarochara fenyesi

XML Treatment for Crataraea suturalis

XML Treatment for Dexiogyia angustiventris

XML Treatment for Ilyobates bennetti

XML Treatment for Ocyusa canadensis

XML Treatment for Oxypoda rubescans

XML Treatment for Parocyusa americana

XML Treatment for Parocyusa fuliginosa

XML Treatment for Brachyusa helenae

XML Treatment for Gnypeta helenae

XML Treatment for Gnypeta nigrella

XML Treatment for Myllaena cuneata

XML Treatment for Myllaena potawatomi

XML Treatment for Agaricomorpha websteri

XML Treatment for Eumicrota corruscula

XML Treatment for Eumicrota socia

XML Treatment for Euvira micmac

XML Treatment for Gyrophaena affinis

XML Treatment for Gyrophaena antennalis

XML Treatment for Gyrophaena brevicollis

XML Treatment for Gyrophaena caseyi

XML Treatment for Gyrophaena criddlei

XML Treatment for Gyrophaena dybasi

XML Treatment for Gyrophaena fuscicollis

XML Treatment for Gyrophaena gilvicollis

XML Treatment for Gyrophaena meduxnekeagensis

XML Treatment for Gyrophaena modesta

XML Treatment for Gyrophaena neonana

XML Treatment for Gyrophaena stroheckeri

XML Treatment for Gyrophaena uteana

XML Treatment for Leptusa carolinensis

XML Treatment for Phanerota fasciata

XML Treatment for Phymatura blanchardi

XML Treatment for Thecturota pusio

XML Treatment for Placusa incompleta

XML Treatment for Placusa vaga

XML Treatment for Acrotona smithi

XML Treatment for Acrotona subpygmaea

XML Treatment for Alevonota gracilenta

XML Treatment for Aloconota sulcifrons

XML Treatment for Atheta capsularis

XML Treatment for Atheta (Atheta) aemula

XML Treatment for Atheta (Atheta) borealis

XML Treatment for Atheta (Atheta) circulicollis

XML Treatment for Atheta (Datomicra) particula

XML Treatment for Atheta (Dimetrota) burwelli

XML Treatment for Atheta (Dimetrota) campbelli

XML Treatment for Atheta (Dimetrota) pseudocrenuliventris

XML Treatment for Atheta (Dimetrota) terranovae

XML Treatment for Atheta (Metadimetrota) savardae

XML Treatment for Atheta (Microdota) alesi

XML Treatment for Atheta (Microdota) festinans

XML Treatment for Atheta (Pseudota?) nescia

XML Treatment for Callicerus obscurus

XML Treatment for Callicerus rigidicornis

XML Treatment for Dinaraea backusensis

XML Treatment for Mocyta breviuscula

XML Treatment for Philhygra jarmilae

XML Treatment for Philhygra laevicollis

XML Treatment for Philhygra luridipennis

XML Treatment for Philhygra proterminalis

XML Treatment for Stethusa klimschi

XML Treatment for Stethusa spuriella

XML Treatment for Strigota ambigua

XML Treatment for Strigota obscurata

XML Treatment for Trichiusa compacta

XML Treatment for Trichiusa hirsuta

XML Treatment for Trichiusa robustula

XML Treatment for Zyras planifer
